# Transcription factor TFII-I fine tunes innate properties of B lymphocytes

**DOI:** 10.3389/fimmu.2023.1067459

**Published:** 2023-01-23

**Authors:** Amit Singh, Mary Kaileh, Supriyo De, Krystyna Mazan-Mamczarz, Dashzeveg Bayarsaihan, Ranjan Sen, Ananda L. Roy

**Affiliations:** ^1^ Laboratory of Molecular Biology and Immunology, National Institutes of Health, National Institute on Aging, Baltimore, MD, United States; ^2^ Laboratory of Genetics & Genomics, National Institutes of Health, National Institute on Aging, Baltimore, MD, United States; ^3^ Center for Regenerative Medicine and Skeletal Development, Department of Reconstructive Sciences, University of Connecticut Health Center, Farmington, CT, United States

**Keywords:** B cell, Gtf2i, FO and MZ B cells, innate and adaptive immunity, chromatin accessibility

## Abstract

The ubiquitously expressed transcription factor TFII-I is a multifunctional protein with pleiotropic roles in gene regulation. TFII-I associated polymorphisms are implicated in Sjögren’s syndrome and Lupus in humans and, germline deletion of the *Gtf2i* gene in mice leads to embryonic lethality. Here we report a unique role for TFII-I in homeostasis of innate properties of B lymphocytes. Loss of *Gtf2i* in murine B lineage cells leads to an alteration in transcriptome, chromatin landscape and associated transcription factor binding sites, which exhibits myeloid-like features and coincides with enhanced sensitivity to LPS induced gene expression. TFII-I deficient B cells also show increased switching to IgG3, a phenotype associated with inflammation. These results demonstrate a role for TFII-I in maintaining immune homeostasis and provide clues for *GTF2I* polymorphisms associated with B cell dominated autoimmune diseases in humans.

## Introduction

TFII-I was discovered as a transcription factor that bound to the adenovirus major late core promoter Initiator (Inr) element. TFII-I also interacts with a sequence-specific DNA element (the E-box element) together with the upstream stimulatory factor (USF) in cell-free systems (1). Subsequent studies have suggested a broader, multifunctional role for TFII-I in a variety of cell types perhaps by virtue of its interaction with cell type-specific proteins and signaling intermediates (2). TFII-I is shown to be phosphorylated in response to several cell surface receptor signaling pathways, including growth factor receptor and immune receptors (3), further suggesting its role in these pathways. Recent studies also indicate its involvement in DNA-damage and response pathways (4).

TFII-I is vertebrate-specific and encoded by the *GTF2I* gene in humans and *Gtf2i* in mice (5). The locus comprises 36 exons that give rise to several alternatively spliced isoforms (6). Various disease pathologies are associated with either gene dosage effects or mutations or gene fusion events involving *GTF2I* (7). Most notably, *GTF2I* haploinsufficiency is associated with Williams-Beuren Syndrome (WBS) a neurodevelopmental disorder with specific craniofacial features (7–9). *GTF2I* gene dosage is further ascribed to autism spectrum disorder (ASD) (10) and single nucleotide polymorphisms in *GTF2I* loci are associated with autoimmune disorders like SLE, RA and Sjogren syndrome (11, 12), and reviewed in (3). Finally, a single point mutation in *GTF2I* is associated with thymic epithelial tumors as well as *GTF2I* gene fusions are known to occur in various forms of cancers (13, 14). Collectively, these studies clearly point to an essential and important role for TFII-I in various human diseases. Consistent with pleiotropic roles of TFII-I, germline deletion of the gene *Gtf2i* in mice leads to early embryonic lethality likely due to severe defects in vasculogenesis and angiogenesis (15, 16).

Although ubiquitously expressed in most if not all cell types, a series of studies showed that TFII-I biochemically interacts with Bruton’s tyrosine kinase (Btk) in B cells and corresponding kinase Itk in T cells, suggesting a role in immune cell type specific functions (17). Moreover, biochemically TFII-I interacts with the B-cell specific co-activator, OCA-B (18) and B cell transcription factor Bright (19) and regulates Immunoglobulin gene expression in cell-based assays (18). Intrigued by TFII-I’s potential involvement in the immune system, we conditionally deleted the *Gtf2i* gene in mice using a CD19-Cre driver to test its potential role in mature B cells.

Murine naïve B cells are generally classified into three distinct subsets, B-1 B cells of peritoneal origin, follicular (FO) B cells, and marginal zone (MZ) B cells (20). A large fraction of FO B cells is IgD^hi^IgM^low^CD21^mid^ cells (termed as follicular type I B cells), while a smaller fraction are IgD^hi^IgM^hi^CD21^mid^ B cells (termed as follicular type II B cells) (21). In contrast, only a minor population of splenic B cells are MZ cells expressing high levels of IgM, CD21 and CD1d. MZ B cells are generated as naive B cells that intrinsically have some properties resembling those of memory cells. MZ B cells are also considered to be innate-like cells that can be induced to differentiate into short-lived plasma cells in the absence of BCR ligation (22). These properties allow the MZ B cells to crossover between adaptive and innate immunity (23).

We show here that ablation of TFII-I in B cell lineage selectively reduces murine MZ B cells. Further, transcriptomic and chromatin studies show that the surviving MZ B cells as well as FO B cells exhibit enhanced innate cell-like molecular features. Consistent with this notion, the splenic B cells in TFII-I deleted mice exhibit a noticeable increase in lipopolysaccharide (LPS) sensitivity, once again resembling features that are characteristic of innate immune cells. These results demonstrate that TFII-I is critical for maintaining B cell homeostasis and its absence accentuates innate properties of B lymphocytes.

## Results

### Reduced numbers of MZ B cells in *Gtf2i^fl/fl^CD19-Cre^+^ (Gtf2i* cKO) mice

**Figure 1:**
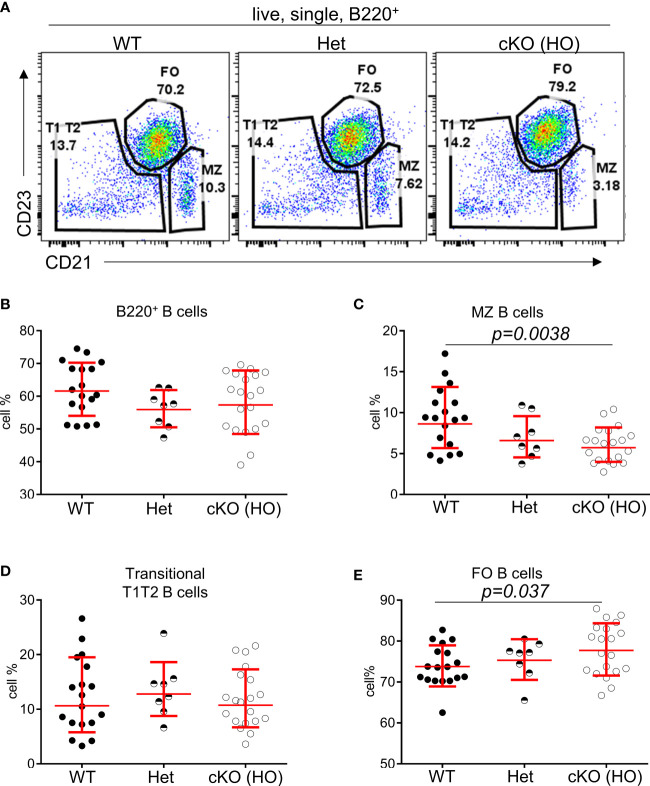
Reduced percentages of MZ B cells in *Gtf2i* cKO mice. **(A)** Representative plots of splenic B cell immunophenotyping of WT, *Gtf2i^fl/+^CD19-Cre+* (Het) and *Gtf2i^fl/fl^CD19-Cre+* (HO) mice. FACS dot plots of CD21 (x axis) and CD23 (y axis) expression in B cells show CD21^lo^CD23^hi^ Follicular (FO) B cells, CD21^hi^CD23^lo^ Marginal Zone (MZ) B cells and CD21^lo^CD23^lo^ Transitional (T1T2) B cells. **(B)** Splenocytes of wild type (WT), heterozygous (Het) and homozygous null (HO) mice were stained with B220 antibody and the percentages of B220+ total B cells are shown. **(C–E)** Primary splenic B cells were isolated from WT, Het and HO mice using EasySep magnetic purification techniques by negative selection. About 1 million B cells were stained with anti CD21-FITC and anti CD23-PE for 35 minutes, then washed and analyzed at the BD FACSAria Fusion. The percentages of FO, MZ and T1T2 B cells from 18 - 20 independent experiments are shown. Significance (P values) were calculated using paired student’s *t* test in Graphpad Prism.

To interrogate the role of TFII-I in B cell function we generated a “conditional knock-out” mouse model by breeding ‘floxed’ alleles of *Gtf2i* (24) with B cell-specific CD19-driver Cre. The B cell specific deletion of *Gtf2i* at genomic, mRNA and protein level were confirmed (**Supp Figure 1A, B**). Total splenocytes and B cell numbers were unaffected upon *Gtf2i* ablation (**Supp Figure 1C**). Various splenic B cells subsets (transitional, FO and MZ) were identified by previously described flow cytometry gating strategy (20) (**Figure 1A**), the percentages of total B, FO, MZ and transitional B (T1, T2) cells from 18 - 20 independent experiments are shown in (**Figures 1B-E**) and the cell numbers from 4 independent experiments are shown in (**Supp Figure 1D**). Both percentages and numbers of MZ B cells were significantly reduced in *Gtf2i* cKO (homozygous, HO) mice compared to control (wild type, WT and heterozygous, Het) mice (**Figure 1C** and **Supp Figure 1D**). However, total B cells as well as the FO and transitional B cell percentage and numbers were comparable among control and cKO mice (**Figures 1B, D, E** and **Supp Figure 1D**).

We further investigated the tonic signaling (low level of antigen independent BCR signaling responsible for B cell survival) (25) in total splenic B cells in absence of TFII-I, as it can have Btk mediated B cell survival effects (26, 27). B cells were cultured *ex vivo* with or without B cell activating factor (BAFF) for the indicated times (**Supp Figure 1E**) and viability was checked using flow cytometry. However, there was no appreciable difference in the viability of B cells with or without BAFF among the WT, Het, and HO mice, suggesting no obvious role of TFII-I in B cell survival under these experimental conditions. Taken together these results indicate that although B cell specific ablation of *Gtf2i* does not interfere broadly with peripheral B cell homeostasis and survival, it selectively reduces MZ B cells in the spleen.

### Effects of *Gtf2i* ablation on B cells transcriptome

**Figure 2:**
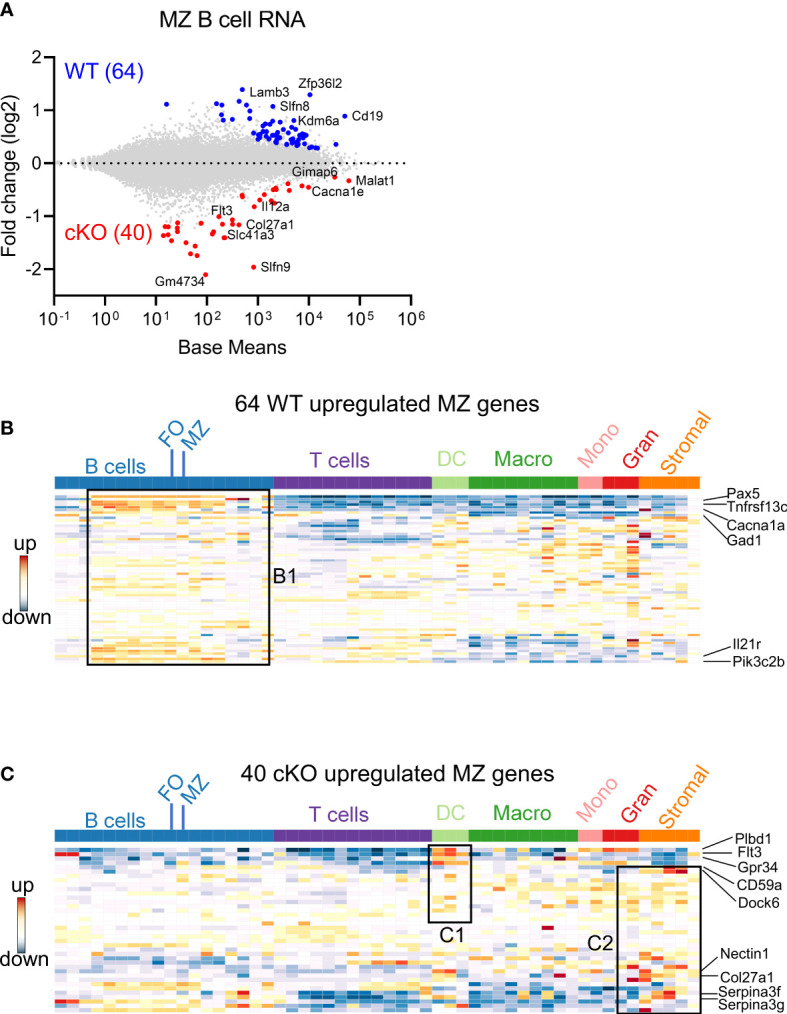
Differentially expressed genes in FO and MZ B cells derived from *Gtf2i* cKO and WT mice. Total RNA was extracted from FO and MZ B cells of WT and cKO mice. The RNA was used for bar-coded library preparations and sequencing. **(A)** MA plot displaying the log fold-change compared with mean expression and showing the DEGs of the WT MZ B cells (64 genes) and of the cKO MZ B cells (40 genes). **(B)** 64 WT MZ specific genes were queried against the ImmGen-Database and the heatmap shows the expression levels of these genes in B and other immune cells. Box B1 shows genes that are significantly enriched in the different B cell compartments. **(C)** Heatmap shows the expression levels of the 40 cKO MZ specific genes when they were queried against the ImmGen-Database. Genes that are enriched in DC compartment are shown in box C1 while genes that are enriched in stromal and granulocytes are shown in box C2.

To understand the possible mechanisms of peripheral differences in B cell subsets, we first analyzed the transcriptome using bulk RNA-seq. Total RNA was prepared from FACS sorted FO and MZ B cells of WT and cKO mice spleens. We first identified MZ B cells differentially expressed genes (DEGs) between *Gtf2i* cKO and WT mice *via* DESeq2 algorithm taking ≥1.5 fold and FDR≤ 0.05 as cutoff (**Figure 2** and **Supp Figure 2A**). DEG numbers were low, 64 DEGs were unique to WT and 40 genes were specific to *Gtf2i* cKO MZ B cells (**Figure 2A**). Of these small number of genes, we noticed *Zfp36l2* was selectively downregulated in cKO MZ B cells. Zfp36l2 belongs to RNA binding protein (RBP) family which is involved in MZ B cell maintenance (28) and regulation of immune inflammation (29). This may partially provide an explanation as to why the cKO mice have reduced numbers of MZ B cells. Whether *Zfp36l2* is a target of TFII-I is presently unknown. Next, we similarly identified FO B cells DEGs between the cKO and WT mice and only 6 DEGs were WT specific and 22 were cKO specific (**Supp Figure 2A, B**).

Overlapping FO and MZ DEGs of cKO B cells identified TFII-I target genes, although at this stage we don’t know whether these are direct or indirect targets. Out of 62 cKO DEGs (40 MZ and 22 FO), 12 genes were shared by both FO and MZ subsets, 10 were FO specific and 28 were MZ specific genes (**Supp Figure 2C**). We noticed that these cKO-specific genes further exhibited specific innate immune related features (**Supp Figure 2C**, boxes bold fonts). This observation led us to further analyze MZ DEGs for presence of potential broad immune cell features using Immunological Genome Database (ImmGen-Database) (30). Genes which were up-regulated in the absence of TFII-I (40 genes) demonstrated enrichment only in MZ B cells (**Supp Figure 2D, E**, box A), while genes which were downregulated in the absence of TFII-I (64 genes) were enriched in the MZ subset as well as in other peripheral B cell subsets, which included mainly the transitional B cells genes like *Pax5*, *Tnfrsf13c*, *Cacna1*, *Gad1*, *Il21r* and *Pik3c2b* (**Figure 2B** box B1 and **Supp Figure 2D**, box A). Interestingly, ImmGen database comparison indicated that some of these 40 cKO genes are also highly expressed in two splenic dendritic cell subsets of bone marrow origin (CD4^+^ and CD8^+^ DCs) genes like *Plbd1*, *Flt3* and *Gpr34* (31, 32) and normally not expressed in splenic B cells (**Figure 2C** box C1 and **Supp Figure 2D**, box B). Further, they exhibited genes such as *Cd59a, Dock6, Nectin1, Col27a1, Serpina3f* and *Serpina3g* (**Figure 2C** box C2 and **Supp Figure 2D**, box C) which come from stromal subsets of Thymic Medullary Epithelial Cells “TEC”, fibroblastic reticular cells “FRC”, lymphatic endothelial cells “LEC” and also represent genes involved in myeloid development (33–35). Together, these observations indicate that although *Gtf2i* deletion in B cells doesn’t make significant transcriptome alteration, it leads both FO and MZ subsets to upregulate selective genes that are of innate and stromal cell origin.

### FO and MZ B cell defining transcriptome in WT and *Gtf2i* cKO mice

**Figure 3:**
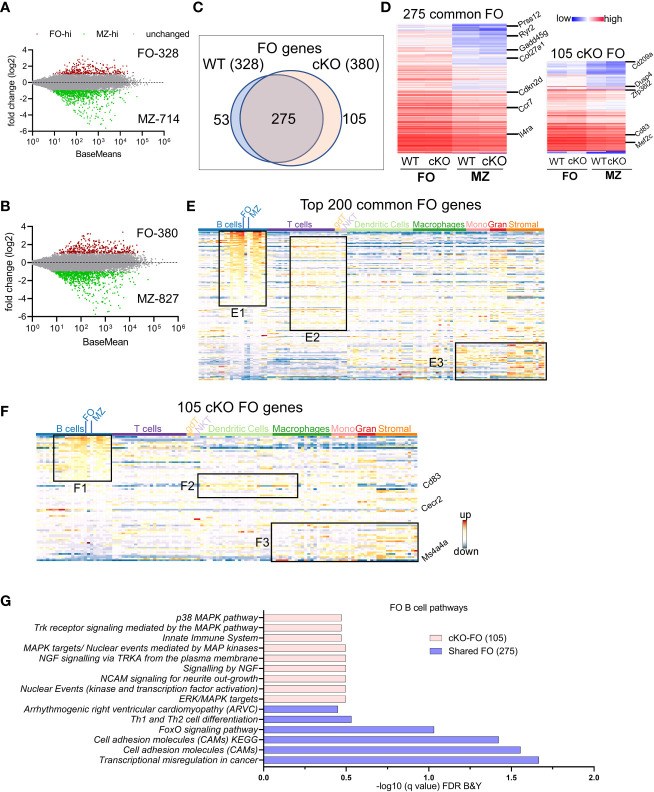
FO and MZ B cell defining transcriptome in WT and cKO mice. Total RNA was extracted from FO and MZ B cells of WT and cKO mice. The RNA was used for bar-coded library preparations and sequencing. **(A, B)** MA plot displaying the log fold-change compared with mean expression and showing the differentially expressed genes (DEGs) in FO and MZ B cells of the WT **(A)** and of the cKO **(B)** mice. **(C)** Venn diagram showing the genes that are common verses the ones unique to FO B cells between the WT and the cKO mice. **(D)** Heatmap demonstrating average counts (CPM, n=2) normalized by DESeq2 of the 275 FO common genes (left panel) and of the 105 unique cKO genes (right panel). **(E)** 200 common FO genes were queried against the ImmGen-Database to identify their enrichment in the different immune cell populations. Heatmap representing the expression of these genes in all immune cells. Genes enriched in the B cell compartments are shown in box E1, genes enriched in T cells compartments are shown in E2 and genes enriched in monocytes, granulocytes and stromal cells are shown in E3. **(F)** 105 cKO FO specific genes were queried against the ImmGen-Database as in E and the genes enriched in B cell compartments are shown in box F1, genes enriched in the Dendritic and Macrophage cells are shown in box F2 while genes that are enriched in the myeloid and stromal lineages are shown in box F3. **(G)** Gene Ontologies (GO) analysis using ToppGene suite, the analysis was carried out using either the shared (shared between WT and cKO) FO or the cKO specific FO B cell genes.

Both follicular and marginal zone B cells develop from transitional B cells and attain their signature genes (20). Since marginal zone cells were reduced in the absence of TFII-I we tested whether TFII-I could alter the signatures of FO and MZ defining gene expression. Using DeSeq2 algorithm, we first identified DEGs between our WT (C57BL6) FO and MZ B cells (**Supp Figure 3A**) and then compared with Immgen-Database C57BL6 FO-MZ DEGs. The analysis of our WT B cells identified 328 upregulated genes in FO B cells and 714 genes in MZ B cells (**Figure 3A**). These numbers were similar to ImmGen-Database (30) when FO-MZ DEGs were compared, indicating a robustness in our analysis (**Supp Figure 3B**). We next assessed the FO and MZ signature DEGs in the absence of TFII-I using cKO mice (**Supp Figure 3A**). *Gtf2i* ablation shows slightly more DEGs between FO and MZ B cells comparable to the WT; 380 genes in the cKO FO cells, while 827 genes in the cKO MZ cells (**Figure 3B**). To determine the features associated with these cKO-specific genes, they were compared to WT using Venn overlap in both FO and MZ subsets.

*FO B cell genes*: For FO specific genes, majority (275) were commonly upregulated (unaltered by *Gtf2i* absence) in both groups (**Figure 3C**) with a similar expression level (**Figure 3D** left panel and **Supp Table 1**). Of these genes *Prss12*, *Ryr2*, *Il4ra*, and *Gadd45g* are known to be FO specific (36). Top 200 common FO genes were analyzed using ImmGen-Database for immune-related transcriptomic signatures, including B cell, T cell, dendritic cells (DC), macrophages (Macro), monocytes (Mono), granulocytes (Gran) and stromal cells (**Supp Figure 3C**). These genes demonstrated features consistent with FO B cell specific genes (**Supp Figure 3C** upper panel red arrow). Interestingly, nearly half of these common FO genes were also significantly enriched in other peripheral subsets of B cells except MZ, germinal center (GC) and peritoneal cavity B cells (**Figure 3E**, box E1 and **Supp Figure 3D**). This analysis reiterates that transcriptionally FO B cells are significantly far from MZ, and GC B cells compare to transitional B cells. A subset of these common genes was expressed to a lower extent in mature T cell subsets (**Figure 3E**, box E2 and **Supp Figure 3D**), while the genes that are expressed at lower levels in FO B cells were upregulated in some subsets of macrophages, monocytes, and stromal cell (**Figure 3E**, box E3 and **Supp Figure 3D**). However, in the absence of *Gtf2i*, 105 genes were upregulated in FO B cells, including *Zfp36l2*, *Dusp4*, *Cd83*, *Cd209a* and *Mef2c* (**Figure 3D** right panel and **Supp Table 2**). Similarly, 53 genes were downregulated in *Gtf2i* ablated FO B cells (upregulated in WT FO B cells) including *Dab2ip* and *Abca8b*. Immgen-Data base comparison of these 53 genes shows that they were also expressed in other B cell subsets (**Supp Figure 3E**, box SE1), including *Vpreb3* (a gene expressed during B cell development) (37). The poorly expressed FO WT genes were expressed in other myeloid and stromal cell compartments (**Supp Figure 3E**, box SE2), a feature observed in the common set of FO genes (**Figure 3E**, box E3). Because these genes were expressed in the absence of *Gtf2i* we concluded that these could be TFII-I (direct or indirect) regulated genes.

Genes upregulated in FO B cells in the absence of *Gtf2i* (105 cKO) were also significantly expressed (**Supp Figure 3D**) in other B cell subsets (**Figure 3F**, box F1). For instance, a Pax5 target gene *Cecr2*, which is expressed during B cells development (38) is still expressed in FO B cells in the absence of *Gtf2i* (**Figure 3F**). In contrast and unlike common and WT FO genes, the FO genes that are specific to the cKO group showed some enrichment of dendritic and macrophage subset genes such as *Cd83* (**Figure 3F**, box F2). Further, the genes that are expressed at low levels in the cKO FO subset were also expressed in other myeloid and stromal cell lineages (**Figure 3F**, box F3). The enriched Gene Ontologies (GO) of the 275 common FO genes were identified using ToppGene suite and demonstrate mostly cell adhesion features (**Figure 3G**). cKO specific 105 FO genes show enrichment of multiple MAPK pathways and innate immune system GOs (**Figure 3G**). These observations indicate that in the absence of TFII-I, and in comparison, to the MZ B cells, FO B cells maintain expression of genes that are highly expressed during B cell developmental stages (*Vpreb3* and *Cecr2*) and genes which mark GC B cells or innate immune cells like *Cd83* and *Cd209a*.

**Figure 4:**
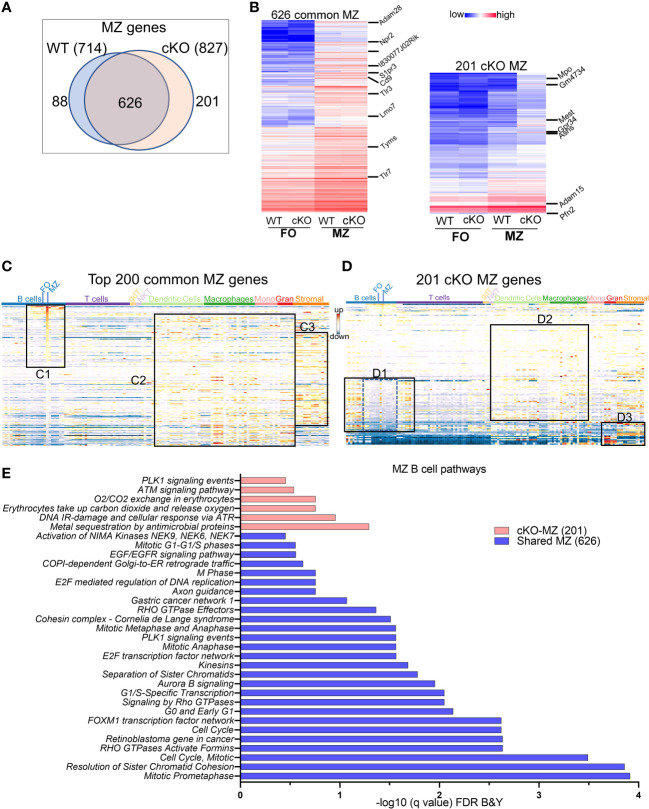
MZ B cell defining transcriptome in WT and cKO mice. Total RNA was extracted from MZ B cells of WT and cKO mice. The RNA was used for bar-coded library preparations and sequencing **(A)** Venn diagram showing the genes that are common verses the ones that are unique to MZ B cells between the WT and the cKO mice. **(B)** Heatmap demonstrating average counts (CPM, n=2) normalized by DESeq2 of the 626 MZ common genes (right panel) and of the 201 unique cKO genes (left panel). **(C)** Top 200 common MZ genes were queried against the ImmGen-Database and the heatmap representing the expression of these genes in all immune cells. Box C1 shows the genes that are enriched mostly in the MZ compartment, box C2 shows the genes that are enriched in the innate compartment and box C3 shows the genes that are enriched in the granulocyte and stromal cell compartments. **(D)** 201 cKO MZ genes were queried against the ImmGen-Database as in **(C)** and the genes that are enriched in B and T cells compartments are shown in box D1, while the genes that are enriched in Dendritic cells and Macrophages are shown in box D2 and the genes that are enriched in myeloid and stromal lineages are shown in box D3. **(E)** Gene Ontologies (GO) analysis using ToppGene suite, the analysis was carried out using either the shared (shared between WT and cKO) MZ or the cKO specific MZ B cell genes.

*MZ-specific genes*: We next compared the transcriptomic features of MZ B cell genes that are enriched when compared to FO B cells in WT and in cKO. The overlapping of 714 upregulated MZ specific genes in WT with 827 genes in *Gtf2i* cKO resulted in 626 common set of genes (**Figure 4A**) that also exhibited comparable expression levels (**Figure 4B**, left panel and **Supp Table 3**). When analyzed for gene features of other immune cells, top 200 common MZ specific genes showed significant (**Supp Figure 3F**) dominating MZ B cell features (**Figure 4C**, box C1). These common genes also recapitulated features of innate immune cell genes (36) (**Figure 4C**, box C2 and **Supp Figure 3F**) and shared features characteristic of granulocytes and stromal cell compartments (**Figure 4C**, box C3). While 201 MZ genes were uniquely upregulated in the absence of *Gtf2i* (*Gtf2i* cKO MZ cell genes) when compared to cKO FO B cells, which include *Mpo*, *Gm4734*, *Mest*, *Gpr34* and *Asns* (**Figure 4B**, right panel and **Supp table 4**). Likewise, 88 MZ genes were downregulated in *Gtf2i* lacking B cells (upregulated in WT MZ cell genes) including *Zfp385a*, *Gap43*, and *E2f7*. WT MZ-specific genes (88 genes) didn’t demonstrate any significant features for other immune cell types (**Supp Figure 3G**). However, the 201 genes derived from the MZ compartment of cKO demonstrated significant enrichment of genes belonging to developing stages of both B and T lymphocytes (**Figure 4D**, box D1 and **Supp Figure 3H**). These genes were generally poorly expressed in mature peripheral lymphocytes of WT mice (**Figure 4D**, box D1, middle region dotted lines) and are mainly cell cycle related genes (**Supp Table 4**). Remarkably, in the absence of *Gtf2i*, MZ high expressing genes further exhibited features resembling innate immune compartments (**Figure 4D**, box D2 and **Supp Figure 3H**). Additionally, cKO MZ genes also showed commonality with granulocyte and stromal cell genes, which are generally not expressed in WT MZ B cells (**Figure 4D**, box D3). Like FO B cells, these observations too suggest that there is an alteration of MZ defining basal transcriptome in the absence of TFII-I. The enriched GOs of the 626 common MZ genes identified mostly cell cycle enriched pathways (**Figure 4E**). While GO analysis for *Gtf2i* cKO specific 201 MZ genes included the PLK1 signaling that has a role in the progression of mitosis, the ATM and ATR signaling pathways that are crucial for DNA damage response and maintains genomic integrity (**Figure 4E**).

In summary, these data demonstrate that although in the absence of TFII-I the overall transcriptomic features distinguishing FO and MZ are largely retained, FO B cells lacking TFII-I exhibit signatures that skew them towards DC and macrophage subsets and have some features of GC B cells. In contrast, MZ B cells gained expression of cell cycle-related genes from developing stages of lymphocytes and further exhibit enhanced signatures characteristic of innate cells.

### Chromatin landscape of FO and MZ B cells in WT and *Gtf2i* cKO

**Figure 5:**
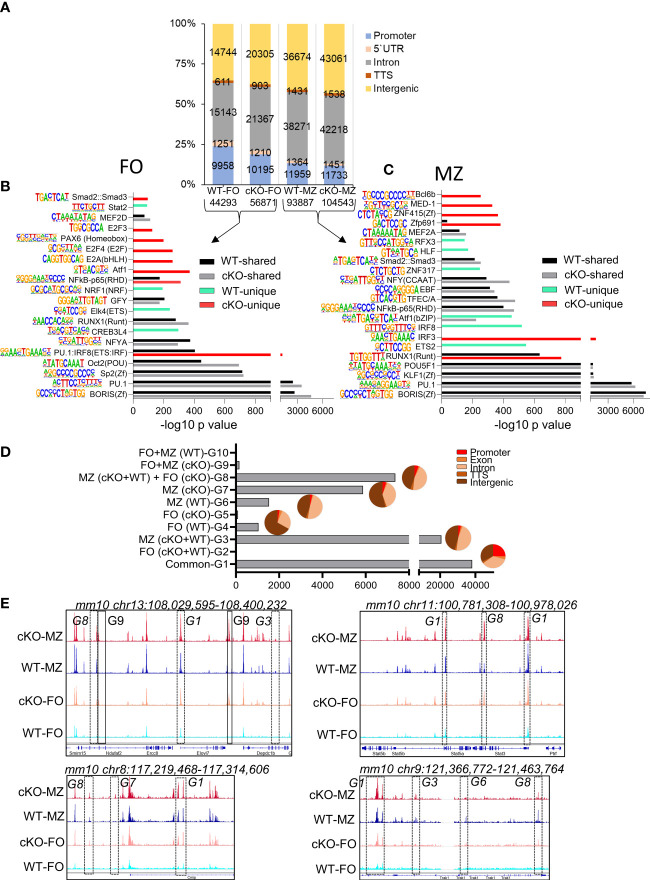
Chromatin landscape of FO and MZ B cells of WT and cKO mice. Quantitative description of chromatin landscape of four different splenic B cell populations: WT FO and MZ B cells and *Gtf2i* cKO FO and MZ B cells. **(A)** Peaks from FO and MZ B cells (numbers of peaks on bottom) from the indicated mouse strain and the annotations regarding their location in the genome. The peak numbers per region are shown inside each region **(B, C)** Presence of transcription factor motifs in open chromatin of WT and cKO FO **(B)** and MZ B cells **(C)**, bars represent the -log10 p-value. cKO selective transcription factor motifs are marked by red color columns while WT selective transcription factor motifs are marked by light green color columns. Motif logos are listed next to each transcription factor. **(D)** Overlapping peaks from all four populations were identified using mergePeaks.pl suite (with -d 50) in HOMER. Ten different clustered groups (G1 – G10) of the overlapping peaks are shown with their indicated properties on the y axis. The pie charts show the peaks distribution in the genomic regions for the specified groups. **(E)** Genomic tracks of open chromatin (snapshot from IGV genome track viewer) from indicated B cell populations demonstrating open chromatin regions of chromosome 13, 11, 8 and 9. Group specific peaks are outlined by boxes.

Although TFII-I is a transcription factor that functions *via* cis-regulatory elements and expected to impact chromatin accessibility, this has not been directly explored. Open chromatin impressions are associated with cellular identities (39). Thus, to address whether absence of TFII-I would alter chromatin landscape of FO and MZ B cells, we performed Assay for Transposase-Accessible Chromatin with high-throughput sequencing (ATAC-seq) (40), using sorted FO and MZ B cells from WT and cKO spleens. After quality checks and alignment to the mouse genome, open chromatin regions (peaks) were called using MACS2 algorithm. We observed substantially fewer peaks in FO B cells in both WT and cKO (44,293 peaks in WT FO and 56,871 peaks in cKO FO) compared to MZ B cells (93,887 peaks in WT MZ and 104,543 peaks in cKO MZ) (**Figure 5A**). Furthermore, we also noted that both FO and MZ B cell subsets of cKO have more open chromatin peaks than WT counterparts, (12,578 and 10,656) respectively. These observations interestingly coincided with the master regulator of chromatin architecture, *Ctcf* mRNA expression levels in FO and MZ B cells. More accessible chromatin of MZ B cells have lower *Ctcf* mRNA compared to FO B cells and similarly cKO FO and MZ B cells also have relatively low expression of *Ctcf* (**Supp Figure 4A**). This result may partially explain the enhanced accessible chromatin peaks observed in cKO B cells (41). Interestingly, a direct interaction between CTCF and TFII-I to regulate gene expression has been observed (42).

To determine where these peaks are located in the genome, we annotated the open chromatin peaks to five distinct genomic regions: promoter, 5`UTR, intron, TTS, and intergenic areas with the number of peaks per region as shown in **Figure 5A**. In the absence of TFII-I the percentage of open promoter regions were reduced in cKO FO B cells with a concomitant gain of open chromatin peaks in the intergenic and intronic regions. Genomic distribution of open chromatin locations in MZ B cells was similar between WT and cKO. We concluded that in the absence of TFII-I the chromatin is more permissive in both FO and MZ splenic B cells and that accessible chromatin is preferentially localized to the intergenic and intronic regions.

We next analyzed RNA corresponding to the accessible chromatin in B cell subsets. Open chromatin peaks were annotated to their associated genes using annotatePeaks.pl algorithm of HOMER. Maximum number of RNA corresponding to the open chromatin (14611) were shared by both FO and MZ subsets of WT and cKO (**Supp Figure 4B**). While MZ B cell subset of both WT and cKO shared next highest number (2476) of open chromatin related genes. Interestingly, there were 1481 open chromatin corresponding genes shared by MZ of both WT and cKO and with FO of cKO (**Supp Figure 4B**). This analysis indicates the possibility that TFII-I might skew cKO FO B cell chromatin accessibility resembling chromatin landscape in MZ B cells.

Open chromatin is often associated with presence or absence of specific transcription factor (TF) or families of TFs binding sites (43). To investigate the specific TF binding sites associated with ATAC-seq peaks, we analyzed the open chromatin of FO and MZ B cells identified in **Figure 5A** using findMotifsGenome.pl algorithm. Similar TF motifs were observed with many open chromatin features associated with WT and cKO derived FO and MZ B cells (**Figures 5B, C**). However, our *de novo* analysis demonstrated IRF8(IRF) motif, which is mainly associated with myeloid and B lymphocyte differentiation (44), was relatively more enriched in FO B cells of cKO as a composite of PU.1:IRF8 (ETS : IRF) shared motif (**Figure 5B**). Interestingly, similar pattern was also observed in MZ B cells of WT (**Figure 5C**). Motifs like IRF8 enriched in WT MZ open chromatin and subtly different motif of IRF3 enriched in cKO MZ open chromatin. Furthermore, RUNX1 motif was enriched in both MZ of cKO and WT with higher enrichment in the cKO MZ than in the WT MZ (**Figure 5C**). Presence of BORIS and PU.1 motifs was quite evident in both FO and MZ cell open chromatin from both strains. The cKO FO and MZ B cell accessible regions additionally exhibited presence of Atf1, E2A, E2F4, NF-κB, Zfp691, ZNF415(Zf), MED-1, and Bcl6b motifs (**Figures 5B, C**). We concluded that though there is more permissible chromatin in the absence of TFII-I, the enrichment of TF-motifs was comparable to WT B cells. However, cKO accessible regions show specific enrichment of E2A and E2F motifs in FO B cells, which can make these cells poised towards GC B cells in the absence of TFII-I (45).

To further understand the global effects of *Gtf2i* ablation on open chromatin landscape across both B cell subsets, we identified common and unique peaks using mergePeaks.pl from HOMER algorithm (46). To reduce redundancy, we considered peaks across the cell types (FO or MZ) or strains (WT or cKO) as same if their start and end sites were within a span of 50 bp. This method provided 10 clustered groups with substantial number of peaks as defined in **Figure 5D**. As expected, the largest number of peaks were common to both cell types (FO and MZ) and strains (WT and *Gtf2i* cKO) as defined in group G1. We hypothesized that these are the open chromatin features of mature splenic B cells. Second largest number of peaks was present in MZ B cells for both strains and is represented by group G3 (20694 peaks). These peaks represented distinct features of MZ cells compared to FO cells regardless of the strain. Furthermore, substantial number of unique peaks (5871) were present in cKO MZ (group G7), suggesting permissive chromatin of MZ B cells in the absence of TFII-I. Surprisingly, apart from these three major groups, 7370 unique peaks were also present in group G8 which is defined as a group consisting of peaks common to both FO and MZ of cKO and MZ of WT. We inferred that even FO B cells partly exhibit open chromatin features like that of MZ B cells in the absence of TFII-I.

Annotation of the common and unique peaks of these groups to the genomic regions is shown in the pie charts in **Figure 5D**. The peaks in group G1 which represent mature B cell open chromatin are almost equally distributed between the promoter (25%), intronic (35%) and intergenic (33%) regions. However, the peaks in the MZ B cells specific group G3 fall mostly in the intronic and intergenic regions with less opening at the promoter region compared to G1. Like G3, the peaks of cKO MZ specific G7 and WT MZ specific G6 fall also mostly in the intronic and intergenic regions. Group G8 which denotes MZ like chromatin of FO B cells in the absence of TFII-I, shows open chromatin mostly in the intronic and intergenic regions, suggesting a role for intronic and intergenic region in regulating the chromatin landscape in these cells. Representative genome browser tracks demonstrating open chromatin peaks from various groups are shown in (**Figure 5E**). Although these open chromatin features suggest that majority of chromatin landscape is not altered by ablation of TFII-I in both FO and MZ B cells, striking differences in selective peaks (as represented by G8) indicate that in the absence of TFII-I, a part of FO B cell open chromatin landscape features begin to skew towards chromatin features resembling that of MZ B cells.

### FO and MZ defining open chromatin features in *Gtf2i* cKO B cells

**Figure 6:**
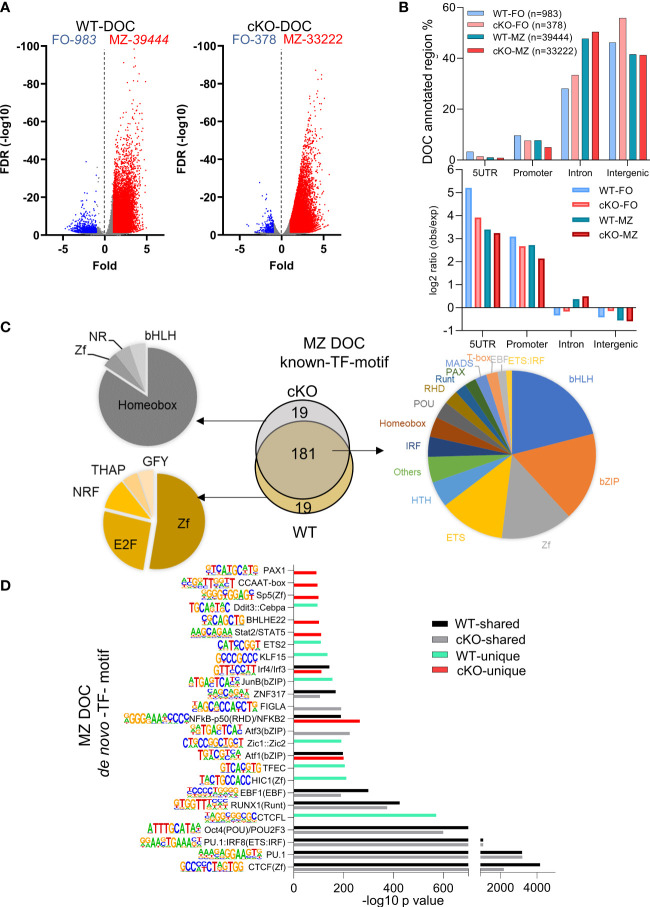
Transcription factors binding motifs in open chromatin of FO and MZ B cells. **(A)** Volcano plot showing the differentially open chromatin (DOC) from FO and MZ B cells of WT (left) and cKO (right); numbers of DOCs per cell type for the WT and cKO are shown on top. **(B)** The DOCs were annotated to the 4 genomic regions (5UTR, promoter, intron, intergenic) and the percentages of these DOC annotated regions are shown for the four cell types. Their observed vs expected values relative to genomic background is shown in bottom. **(C, D)** The top 200 transcription factor (TF) motifs of DOC of MZ from WT and cKO B cells identified by known **(C)** and *de novo* **(D)** TF motif algorithm of HOMER. **(C)** Venn diagram (middle) shows the number of predicted common and unique TF binding motifs. Common motifs are represented by the pie chart on the right while the unique ones are represented on the left side. **(D)** bars represent the -log10 p-value. cKO selective transcription factor motifs are marked by red color columns while WT selective transcription factor motifs are marked by light green color columns. Motif logos are listed next to each transcription factor.

After identifying basal state properties of open chromatin of FO and MZ B cell in the absence of TFII-I, we next analyzed FO and MZ defining open chromatin features in WT and cKO B cells by using DiffBind algorithm (47). Significantly more chromatin peaks were open in MZ B cells compared to FO B cells of both strains, WT (39444 vs 983) and cKO (33222 vs 378) respectively (**Figure 6A**). We further noticed >2fold decrease in differentially open chromatin (DOC) peak numbers in cKO FO B cells when compared to WT FO peaks (378 cKO FO vs 983 WT FO peaks) (**Figure 6A**). When examined for their genomic location annotations, *Gtf2i* cKO FO and MZ specific peaks were reduced at the promoter and 5` UTR region compared to their WT counterpart while there was more cKO FO peaks at the intronic and intergenic region compared to WT FO peaks (**Figure 6B**, top). Relative to the murine genome (mm10) these enrichments were significant for 5` UTR and promoter in both WT and cKO B cells (**Figure 6B** bottom). However, relative to the murine genome, FO B cell, intron and intergenic peak enrichments were less in WT compared to cKO, while MZ B cells appeared to have similar distribution in intron and intergenic region DOCs (**Figure 6B**, bottom). These results indicate that TFII-I ablation also leads to reduced chromatin landscape differences between FO and MZ B cells.

Because of higher number of DOCs, we next focused on MZ specific regions for presence of DEGs association and transcription factor motifs using both known and *de novo* algorithms of HOMER suite. MZ B cell DOCs were annotated to their respective genes and were correlated with DEGs in these cells (**Supp Figure 4C**). Around half of the DEGs (414 genes) have shared DOCs, while the remaining of the genes cannot be associated with differentially open chromatin (**Supp Figure 4C**). By comparing the top 200 transcription factor motifs (identified by known motifs algorithm) of MZ DOC between WT and cKO, 90% (181) of motifs were shared between both genotypes. We also noticed significantly enriched transcription factor motifs of basic-helix-loop-helix (bHLH), basic-leucine zipper (bZIP), zinc finger (Zf), and ETS family in this analysis (**Figure 6C**). In the absence of *Gtf2i*, 19 motifs under MZ DOC were dominated by Homeobox TF motifs while the WT MZ DOC were enriched for Zf and E2F motifs (**Figure 6C**).

In our *de novo* TF motif algorithm analysis (which ranks the dominating TF motifs/TF-family), we found that PU.1, CTCF, POU2F and RUNX1(Runt) motifs were present in open chromatin of both WT and cKO MZ B cells (**Figure 6D**). Interestingly, CTCFL, EBF1, Jun, TFEC and HIC1 motifs were selectively enriched in WT MZ specific open chromatin, while Aft3, Bhleh22, PAX1 and Stat2/STAT5 motifs dominated in the cKO MZ specific open chromatin (**Figure 6D**). Gene expression levels of many of these transcription factors are shown in **Supp Figure 4D**. For example, although Stat family factors show that FO B cells of WT and cKO have similar expression levels, they are higher in the FOs compared to the MZs. On the other hand, *Irf4* shows higher expression in the cKO FO and MZ B cells, while *Irf8* has higher expression levels in FO B cells of WT and cKO. These selective patterns of activated cell and innate like TF-motifs enrichment in differentially open chromatin suggest that the altered chromatin landscape may stem from FO B cell stages itself and the absence of TFII-I may influence all mature B cells.

### Innate response of B cells in absence of *Gtf2i*

**Figure 7:**
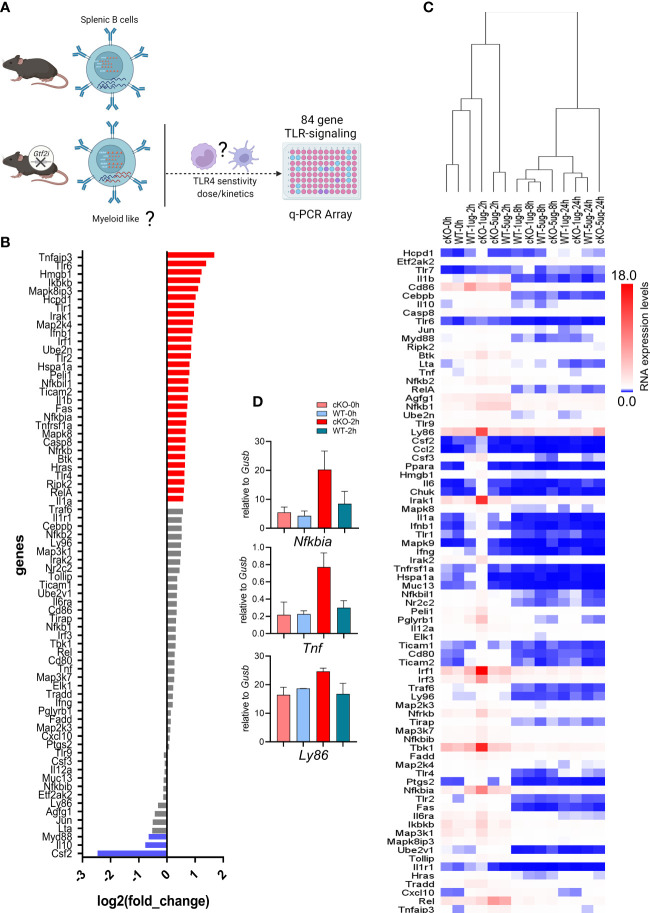
Transcriptome of LPS responsiveness splenic B cells upon *Gtf2i* ablation. **(A)** Scheme representing (created using BioRender.com) the identified differences of transcriptome and open chromatin in B cells upon *Gtf2i* ablation. These differences indicate cKO B cells to possess myeloid like features, which are tested for TLR4 sensitivity by activation with LPS (5 and 1µg/ml) at early, mid and late time points (2, 8 and 24 hours) using TLR-signaling array. **(B)** Basal level expression of genes downstream of TLR-signaling in WT and cKO B cells. Red bars represent 30 genes highly expressed in cKO and blue are highly expressed in WT by at least 1.5-folds (n=4). **(C)** Heatmap representing the RNA expression levels of 84 TLR-responsive genes from LPS high (5µg/ml) and low (1µg/ml) dose at early (2h), mid (8h) and late (24h) time points (n=3). Relative expression of target genes was calculated to geometric mean of Ct values of *Gusb* and *Hsp90a*. **(D)** *Nfkbia, Tnf* and *Ly86* mRNA levels were determined from the Mouse Toll-Like Receptor Signaling Pathway” RT^2^ Profiler PCR Array as in **(C)**, the average of 2 independent experiments is shown. Error bars represent standard deviation.

It is well-established that murine B cells are less responsive to bacterial endotoxin LPS compared to the innate immune cells (e.g., macrophages and dendritic cells) (48). Given the clear indications from transcriptome and chromatin landscape analysis that removal of TFII-I accentuates innate-like features in B cells, we next aimed to test this notion by challenging B cells from WT and cKO *in vitro* with various dosages of LPS and assay for gene expression at various time points using Toll-like Receptor Signaling Pathway RT^2^ Profiler PCR Array (**Figure 7A**). Naïve splenic B cells from WT and cKO mice were stimulated with 1 and 5 µg/ml of LPS for 0, 2, 8 and 24 hours. After RNA extraction and cDNA preparation, samples were subjected to the PCR array analysis. Differential gene expression analysis showed that 36% of the genes (30 genes) of the Toll-like receptor signaling pathway (84 genes) were expressed at higher levels in cKO B cells at basal level itself (**Figure 7B**, red), while three out of the 84 genes exhibited higher expression in WT B cells (**Figure 7B**, blue). These results show that at the basal level most of the Toll-like receptor pathway genes are upregulated in the cKO B cells compared to WT B cells.

Interestingly, the suboptimal dose of LPS (1μg/ml) for B cells shows striking differences at early time point (2h) (Fig 7C - D). In hierarchal clustering the WT B cells (2h, 1µg/ml) clustered with unstimulated B cells (0h) while cKO B cells with the LPS 1μg/ml dose clustered away from WT (1µg/ml) and the unstimulated samples but closer to LPS 5µg/ml dose. We noticed with 1μg/ml dose activation at 2h in cKO B cells some genes, *Ly86*, *Irak1*, *Irf1*, *Tbk1* and *Nfkbia* were induced even higher than 5µg/ml dose. However, the response to optimal dose of LPS (5μg/ml) was similar between the cKO and WT B cells at earlier and late time points (2 and 24 hours) while mid time point (8h) shows some differences (**Figure 7C**). Consistent with the RNA-seq and ATAC-seq analysis, these results together suggest that in the absence of TFII-I, the LPS sensitivity in B cells is increased, further indicating enhancement of their innate and/or lower threshold towards activation.

### Skewed B cell properties in the absence of *Gtf2i*

**Figure 8:**
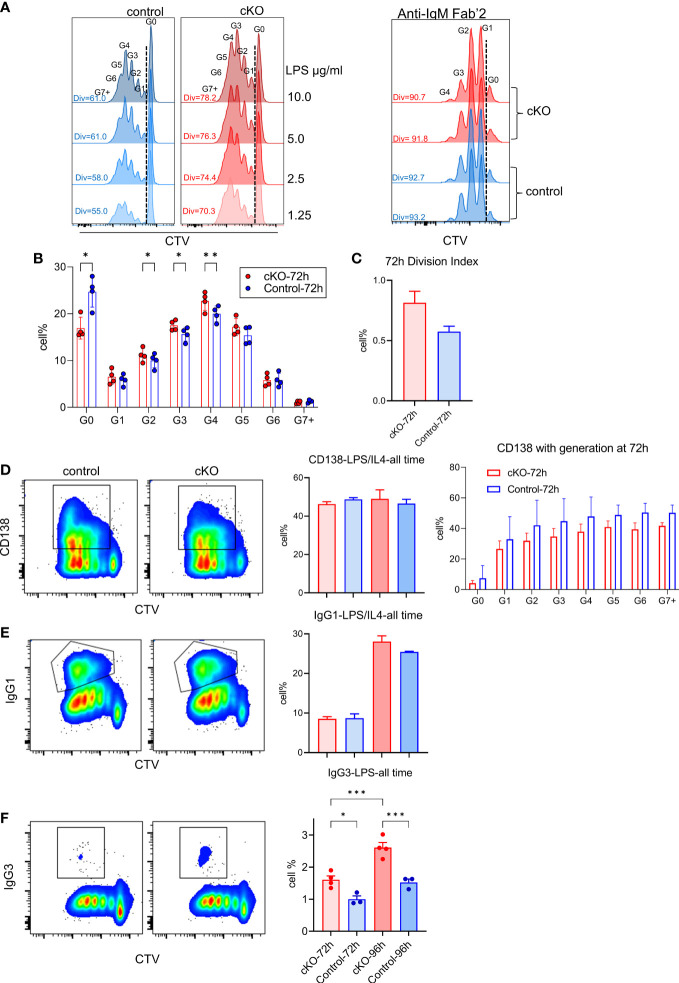
Functional characterization of splenic B cells from WT and cKO derived mice. Splenic B cells from WT and cKO mice were activated with LPS (1.25, 2.5, 5 and 10µg/ml), F(ab`)2 anti-IgM (10µg/ml) or LPS (10µg/ml) plus IL-4 (20ng/ml) for the indicated time points followed by flow cytometry analysis. **(A)** Representative flow cytometry plot of Cell Trace Violet (CTV) dilution after 72 hours of LPS (10µg/ml n = 4, other concentrations n = 2) or anti-IgM (n = 2) activation. The Div numbers indicate the percentages of divided cells for the indicated mouse strain. Numbers above the CTV peaks refer to the cell generation (G0 – G7+) **(B)** Cell percentages at each cell division after 72 hours of LPS activation. Bars represent the mean +/- SEM obtained from 4 independent experiments and p values are shown for paired t-test corrected for multiple comparisons. **(C)** Division Index was calculated for cKO and WT B cells after 72 hours using FlowJo proliferation platform. **(D, E)** Representative flow cytometry plot of CD138 **(D)** or IgG1 **(E)** versus CTV for WT and cKO (left) after 96 hours of LPS plus IL-4 activation. Gating of CD138^+^ and IgG1^+^ cells are indicated. The bar plot on the right shows the average of the CD138^+^ (and the CD138^+^ cell percentages per cell division) or IgG1^+^ cell percentages at the indicated times. **(F)** Representative flow cytometry plot of IgG3 versus CTV after 96 hours of LPS activation and the bar plot shows the IgG3^+^ cell percentages at the indicated time points. Data represent 4 independent experiments *p≤0.05, **p≤0.01, ***p≤0.001.

In the absence of TFII-I in B cells, our transcriptome and chromatin landscape demonstrated skewed properties towards innate like features. Even, FO B cells exhibited enhanced MZ-like molecular features. We thus tested the consequences of such alteration by analyzing ex vivo proliferative properties of B cells after T-independent and dependent (antigen) activation accomplished by LPS and anti-IgM Fab`2 stimulation respectively (**Figure 8**). B cells from *Gtf2i* cKO demonstrated higher proliferation capacities upon LPS-mediated/T-independent stimulation in a dose dependent fashion (1.25, 2.5, 5 and 10ug/ml) particularly at 72h (**Figure 8A** and quantified **Figure 8B**) while there was no difference in proliferation in response to anti-IgM F(ab)`2/T-dependent stimulation (**Figure 8A**). We also noticed that significantly more quiescent B cells progressed into cell cycle and completed first mitotic division in cKO B cells compared to WT B cells upon LPS activation (**Figure 8B**, G0 population). Furthermore, the percentages of cells that proceed to G2, G3 and G4 were significantly higher in cKO B cells compared to the WT (**Figure 8B**). This increased proliferative property after LPS stimulation was further demonstrated by higher division index of cKO B cells at 72h (**Figure 8C**).

To further characterize these B cells functionally, LPS and IL4 treated splenic B cells from WT and cKO mice were tested for CD138^+^ plasma cells differentiation and IgG1 class switching. Although, at total population level CD138^+^ cells were similar between WT and cKO (**Figure 8D**, left), we noticed the trend of reduced plasma cell differentiation at each division in LPS+IL4 treated cKO B cells (**Figure 8D**, right). Similarly, we did not observe any significant difference in IgG1 class switching (**Figure 8E**). Only LPS treated B cells, however, demonstrated switching to inflammation associated IgG3 and was significantly higher in cKO B cells (**Figure 8F**). We concluded that in the absence of *Gtf2i*, splenic B cells become more responsive to innate signals like LPS activation that is reflected in enhanced proliferation and become biased towards class switching to a potently proinflammatory antibody, IgG3 isotype.

## Discussion

Immune responses to pathogens often involve a two-step process: first a nonspecific but rapid defense mechanism of the innate immune system is triggered and subsequently, a specific but slow defense mechanism of the adaptive immune system kicks in. While the innate arm is conserved from drosophila to man, the adaptive arm is exclusively present in vertebrates. These two arms evolved different surface receptors and specialized cells to respond distinctly to different cellular threats and environmental insults *via* triggering distinct intracellular signaling pathways (49). Although there is a boundary between these two arms with distinct cells and signaling pathways mediating such immune responses, murine MZ B cells are ideally located as sentinels at the interface between the circulation and lymphoid tissue to respond to blood-borne pathogens, thereby adopting a hybrid immune strategy that crosses over such boundaries (22). Thus, together with B-1 B cells, MZ B cells are endowed with a “natural memory” that provides a bridge between innate and adaptive immune responses (23). Consistent with this notion, MZ B cells are intrinsically capable of very rapidly maturing into plasmablasts, which may be due, in part, to the ability of MZ B cells to respond quickly than follicular B cells, to both B cell receptor (BCR) and to Toll-like receptors (TLRs) (23). In addition, MZ B cells also express high levels of TLRs (comparable to innate cells like DCs, macrophages and granulocytes). These properties allow the MZ B cells to crossover between the two arms of the immune system and rapidly respond to pathogens both *via* T-independent as well as T-dependent pathways (20, 23).

In this study, we determined that conditional ablation of *Gtf2i* in B cells leads to significant reduction in MZ B cells (Fig 1), without altering the number of splenocytes or B cells. Moreover, there was no appreciable difference in the viability of B cells with or without BAFF between control and *Gtf2i* cKO mice. More surprising is the fact that the remaining MZ B cells as well as the FO B cells derived from *Gtf2i* cKO mice acquired accentuated molecular features of the innate-like properties of MZ B cells. This is reflected in total transcriptomic/RNA-seq (Figs 2-4) as well as chromatin landscape/ATAC-seq analyses (Figs 5 and 6). Although the exact molecular mechanisms for this function of TFII-I is currently unclear, there are several indications from our results. One key molecule may be the RNA binding protein (RBP) family gene *Zfp36l2* that was selectively upregulated in WT MZ B cells, but not in *Gtf2i* cKO MZ B cells (Fig 2A). ZFP36L1 protein post-transcriptionally represses expression of the transcription factors KLF2 and IRF8, which are known to favor the FO B cell phenotype. Given this established role of *Zfp36 family* in MZ B cell maintenance (28), downregulation of *Zfp36l2* might provide a rationale as to why the cKO mice have reduced numbers of MZ B cells. Consistent with this notion, we also noted that genes like *Cd83* which play crucial role in B cell activation and GC B cell responses (50) was upregulated FO cKO B cells. This indicates that in absence of TFII-I B cells may be poised towards activation and can have different kinetics of GC responses. IRF8 motif was significantly more enriched in the open chromatin of the cKO FO B cells (**Figure 5**). Given that TFII-I has been implicated in regulating homeotic genes like *Dlx5* and *Dlx6* (51) our observation that loss of TFII-I results in the open chromatin of homeobox containing motifs (**Figure 6**) also suggests that regulation of homeobox genes in B cell function could be an important role of TFII-I. Further, it can also substantiate the association of homeobox transcription factors with MZ B cell lymphomagenesis (52) thereby indicating a potential role for TFII-I in this process. We noted that differences in B cell chromatin landscape observed upon removal of TFII-I in basal state is far more pronounced than the differences noted with our transcriptomic/gene expression analysis. The incongruence between gene expression and chromatin features has been noted and could be due to a number of factors, including but not limited to the fact that the ATAC-seq assay provides a more dynamic change reflecting the cellular state versus the transcriptomic analysis which is more of a static snapshot (53). But it is also possible that a far more robust difference in gene expression and correlation with open chromatin peaks might be more readily observed in the absence of TFII-I upon antigenic stimulation or infection *in vivo*.

B cell response to LPS is far less robust compared to innate cells, requiring greatly increased dose of LPS to elicit a comparable downstream effect (48). We rationalized that if *Gtf2i* cKO B cells acquired enhanced innate-like features, they might respond to a lower dose of LPS. We found that LPS sensitivity of these cells was also altered (**Figure 7**). In particular, a suboptimal dose of LPS at early time points elicited a robust response in cKO *ex vivo* splenic B cells, compared to the WT control B cells. To our surprise, 5 genes (*Ly86*, *Irak1*, *Irf1*, *Tbk1* and *Nfkbia*) were even more induced with the lower dose of LPS compared to higher dose. This apparently paradoxical regulatory mechanism may be due to time kinetics and/or dose dependent activation of stimulus specific downstream genes *via* enhancer-based mechanisms (54). Further specific studies are required to understand this interesting phenomenon.

To further distinguish functional differences between cKO and WT B cells and considering the fact that *Gtf2i* cKO MZ B cells exhibit enhanced expression of cell cycle genes like developing lymphocytes, we analyzed their proliferative potentials in response to LPS. B cells from *Gtf2i* cKO demonstrated higher proliferation capacities at 72h both at suboptimal and optimal dose of LPS (Fig 8A), while no such appreciable differences were noted when these cells were stimulated using anti-IgM Fab`2. Consistent with this observation, we also noted a far greater number of cells progressed into cell cycle in cKO B cells compare to WT B (**Figure 8B**, G0 population) as demonstrated by higher division index of cKO B cells at 72h (**Figure 8C**). Thus, a TFII-I deficiency in an adaptive immune cell such as B lymphocytes leads to a better proliferation capacity in response to innate signals. Differences in proliferative responses to TLR4 stimulation versus BCR crosslinking also indicate the possibility that TFII-I may play a critical role during T-independent B cell response rather than a T-dependent one. Further, this gained cell number can potentially generate increased immune response, which could be beneficial or deleterious if not regulated properly. Paradoxically, TFII-I is noted to be an oncogene and a gain of function mutation is involved in thymomas and exhibits pro-proliferative capabilities in *in vitro* experiments (14), although proliferative potentials of TFII-I in B cells have never been tested before. Given the distinct and often opposing roles of TFII-I and its isoforms and its different family members in various cell types, further work needs to be done to clearly delineate the function of TFII-I in proliferation in different cell types.

Functional studies of CD138^+^ plasma cell differentiation and Ig class switching upon LPS and IL4 or only LPS stimulation *ex vivo* (Fig 8) further confirm that in the absence of *Gtf2i*, splenic B cells exhibit enhanced responsiveness to LPS by undergoing increased proliferation (a characteristic feature of innate cells), without significantly altering their *in vitro* plasma differentiation properties. But interestingly, *Gtf2i* cKO B cells are clearly biased towards class switching to a potent proinflammatory antibody, IgG3 (55), further suggesting a role for TFII-I in altering B cell differentiation properties. We anticipate that these B cell-specific functions of TFII-I observed under basal conditions will be more pronounced *in vivo* particularly upon T-independent antigenic challenge.

In these studies, we uncovered an unexpected cell-type specific role for TFII-I in murine B cell homeostasis through its conditional ablation. Given that the gain-of-function of TFII-I results in tumorigenic properties, we also conjecture that gain of TFII-I function could lead to B cell specific tumors. We further speculate that the B cell specific functions of TFII-I uncovered here might be related to its known role in autoimmune disorders, like Sjogren syndrome and Lupus. Especially in light of the fact that serum IgG levels (including IgG3) are elevated in Sjogren syndrome and B cell subsets are altered (56), association of these features with lack of TFII-I could explain some aspects of the disease. Finally, although typical WBS syndrome clinical features do not include immune dysregulation, Kimura et al. recently showed differential expression of genes related to B cell activation in WBS patients relative to controls (57). We anticipate future work will address the precise role of TFII-I in autoimmunity and WBS.

## Materials and methods

### Mice

Gtf2i^fl/+^CD19-Cre+ (Het) and Gtf2i^fl/fl^CD19-Cre+ (HO) were generated by crossing Gtf2i^fl/fl^ (24) with Cd19-cre (006785, The Jackson Laboratory). BL6 (WT), Gtf2i^fl/+^CD19-Cre+ (Het) and Gtf2i^fl/fl^CD19-Cre+ (HO) mice were maintained in the animal facility of the National Institute on Aging. Eight to 12 weeks old mice were used for all experiments. The studies were carried out in accordance with the recommendations in the Guide for the Care and Use of Laboratory Animals (NRC 2011). Mice were euthanized with carbon dioxide and spleens were harvested for analysis. The protocol was approved by the Animal Care and Use Committee of the NIA Intramural Research Program, NIH. This program is fully accredited by the Association for Assessment and Accreditation of Laboratory Animal Care International (AAALAC) (File 000401), registered by the United States Department of Agriculture (51-F-0016) and maintains an assurance with the Public Health Service (D16-00602).

### MZ, FO and B cell isolation

Primary B lymphocytes were isolated from spleens of WT, Het and HO mice using EasySep B cell kits by negative selection according to the manufacturer’s instructions (Stemcell Technologies, Canada). B cells were stained with anti CD21-FITC and anti CD23-PE antibodies (Biolegend, San Diego, CA) and sorted at the BD FACS-Aria Fusion. Follicular (FO) B cells were sorted as CD21^lo^CD23^hi,^ and Marginal Zone (MZ) B cells were sorted as CD21^hi^CD23^lo^. Cells were cultured in RPMI 1640 medium (Invitrogen, MA) containing 10% heat inactivated FBS (Gemini Bioproducts, CA), 50μM β-mercaptoethanol (Sigma-Aldrich, MO), 1% L-glutamine and 1% penicillin-streptomycin solution (Gibco, MA). B cell purity was > 95% based on flow cytometric analysis following staining with anti-CD19 (Biolegend, San Diego, CA). FO and MZ B cells were >90% pure based on the criteria that they were sorted on.

### Transcriptomic analysis

Total RNA was extracted from FO and MZ B cells using the RNeasy Mini Kit (Qiagen, Valencia, CA) according to the manufacturer’s protocol. Total RNA was sequenced at the Johns Hopkins Deep Sequencing and Microarray Core using standard protocol for NextSeq 500 sequencer. Ribosomal RNA was depleted, barcoded libraries were made, and 50bp single end reads were generated for each sample. Reads were analyzed using Galaxy RNA-seq pipeline (usegalaxy.org) and adapter trimmed sequences were aligned to mouse genome (mm10) using HISAT2. FeatureCounts and htseq-count were used for differential gene expression analysis using DESeq2. Using “MyGeneset” suite we identified our gene set expression across ImmGen cell-types in immgen.org database (58). Gene ontologies and Immgen-cell type enrichment scores were calculated using ToppFun suite at https://toppgene.cchmc.org/.

### ATAC-seq

FO and MZ splenic B cells were FACS sorted and ATAC-seq libraries were made as previously described (Buenrostro, Giresi et al., 2013) with minor modifications. Briefly, 100,000 cells were lysed in 100 ul of lysis buffer (10 mM Tris-Cl pH 7.4, 10 mM NaCl, 3 mM MgCl_2_, 0.1% NP-40). After centrifuging at 500g for 10 min at 4°C, pelleted nuclei were resuspended with 50 ul of transposition mix (1X Tagment DNA buffer, Tn5 Transposase, nuclease-free H_2_O) and incubated for 30 min at 37°C in a thermomixer with slow revolutions. Transposed DNA was purified using MinElute columns (28004, QIAGEN) and subsequently amplified with Nextera sequencing primers and NEB high fidelity 2X PCR master mix (New England Biolabs) for 11 cycles. PCR-amplified DNA libraries were size selected after fractioning on high melting agarose gel electrophoresis. Library was pooled and sequenced using the Illumina NextSeq sequencer with paired end reads of 75 bases. After sequencing the ATAC-seq analysis was performed on Galaxy server (usegalaxy.org). Reads were run for quality control and treated accordingly for trimming and adapter removing.

### ATAC-seq analysis

QC passed reads, were aligned to mouse mm10 genome using Bowtie2 and peak calling was done by MACS2 peak caller algoritm. HOMER (http://homer.ucsd.edu/homer) software was used for analysis of the open chromatin peaks, mergePeaks.pl (with 50bp overlap to remove redundancy) was used to identify overlapping and unique peaks between various cell subsets. Similarly, within the open chromatin regions findMotifsGenome.pl algorithm was used to identify transcription factor binding motifs. From HOMER suite annotatePeaks.pl algorithm identified genes associated with the peak, location in the genome and observed/expected values of the annotated regions. Differential open chromatin was identified using DiffBind algorithm.

### Activation and gene expression analysis

Splenic B cells were isolated from WT and cKO mice and cultured in presence of LPS (Invivogen, San Diego, CA) 5µg/ml or 1µg/ml for 2, 8 and 24 h time points. Total RNA was extracted from B cells using RNeasy Mini Kit (Qiagen, Valencia, CA). 100ng RNA was reverse transcribed using SuperScript IV VILO Master Mix (Thermofisher Scientific, MA) and the cDNA was loaded onto the “Mouse Toll-Like Receptor Signaling Pathway” RT^2^ Profiler PCR Array according to the manufacturer instructions (Qiagen, Valencia, CA). Expression of the target genes were normalized to the geometric mean of Ct values of the two housekeeping genes *Gusb* and *Hsp90a* using the ddCt method (n = 3). Basal level (0 h) fold change was calculated by dividing the gene expression of cKO B cells on the gene expression of WT B cells (n = 4).

### Proliferation and class switching

Splenic B cells were labeled with Cell Trace Violet (CTV) (Invitrogen, MA) according to the manufacturer instructions. After washing cells were activated with LPS 1.25, 2.5, 5 and 10 µg/ml (10µg/ml n = 4, other concentrations n =2) or with F(ab`)2 anti-IgM 10µg/ml (n = 2) for 72 h followed by flow cytometry analysis for CTV dilution. Each division generation are labeled from G0 – G6. Proliferation plugin of FlowJo (TreeStar BD Biosciences) was used to calculate division and expansion index.

For plasma cell differentiation and IgG1 class switching analysis, LPS/IL4 activated B cells were co-stained with anti-CD138-BV711 and anti-IgG1-PE respectively at 72 and 96 h time points. For IgG3 class switching analysis, LPS activated B cells were stained with anti-IgG3-FITC at 72 and 96 h. Flow acquisition was performed on BD Symphony A5 system and analysis was done on FloJo (TreeStar BD Bioscience).

### Statistics and graphing

Graphad Prism (v9.4.1) was used to perform statistical tests as well as generating graphs for the data. Students t-test was performed as indicated for paired or unpaired tests.

## Data availability statement

The data presented in the study are deposited in the GEO repository, accession number GSE222325.

## Ethics statement

The animal study was reviewed and approved by National Institute on Aging and Animal Care and Use Committee of the NIA Intramural Research Program, NIH.

## Author contributions

AS and MK designed, performed and analyzed experiments. AS and MK share equal first authorship. SD and KM-M analysed RNA-seq and ATAC-seq data. DB generated mouse strain. AS, MK, RS, and AR wrote manuscript. RS and AR secured intramural funding. All authors contributed to the article and approved the submitted version.
