# Supplementary material for: Transcription factor TFII-I fine tunes innate properties of B lymphocytes
Source: Front Immunol. 2023 Jan 23;14:1067459. doi: 10.3389/fimmu.2023.1067459 (PMC9900109; doi:10.3389/fimmu.2023.1067459)
Supplement: Supplementary file 1 [file DataSheet_1.pdf]

Supp. Fig1:

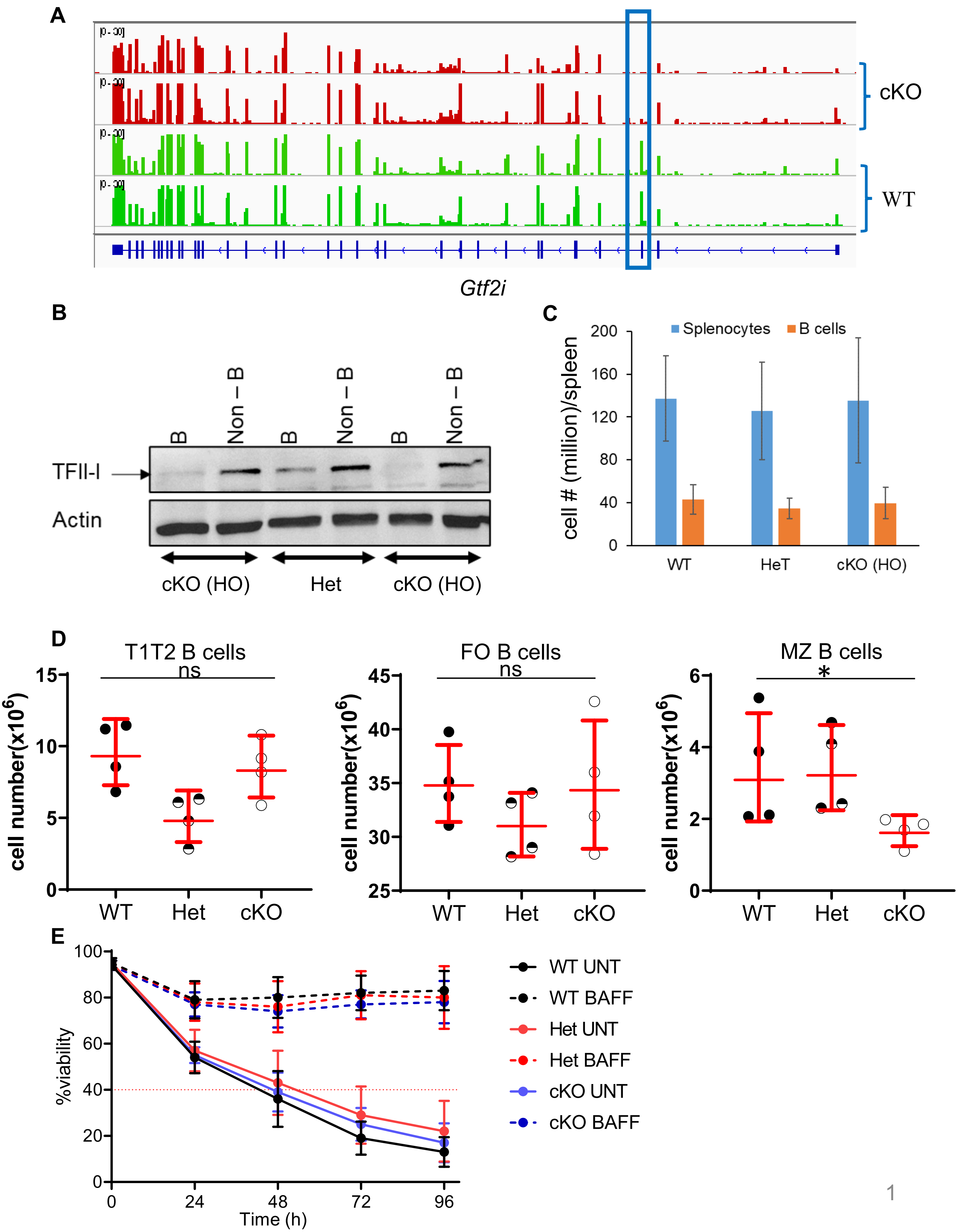
A
cKO
WT
Gtf2i
B
B
Non – B
B
Non – B
B
Non – B
TFII-I
Actin
cKO (HO)
Het
cKO (HO)
C
Splenocytes
B cells
cell # (million)/spleen
200
160
120
80
40
0
WT
HeT
cKO (HO)
D
T1T2 B cells
ns
FO B cells
ns
MZ B cells
*
cell number(x10^6)
15
10
5
0
45
40
35
30
25
6
4
2
0
WT
Het
cKO
E
WT UNT
WT BAFF
Het UNT
Het BAFF
cKO UNT
cKO BAFF
%viability
100
80
60
40
20
0
0
24
48
72
96
Time (h)

Supp. Fig1:

A: Genome Browser tracks (IGV) from the RNA-Seq analysis of *Gtf2i gene in* WT and cKO B cells. Figure shows duplicate samples of each strain. Box represent exon 3 position of the *Gtf2i* gene.

B: Splenic B cells were isolated from the *Gtf2i$^{fl/fl}$CD19-Cre+ cKO (*HO) and *Gtf2i$^{fl/+}$CD19-Cre* Het mice using Easysep B cell isolation kit by negative selection and the rest of the splenocytes were used as control (Non-B cells). B and non-B cells were lysed in RIPA lysis buffer. Whole cell extracts were fractionated by SDS-PAGE and TFII-I protein expression was analyzed by immunoblotting. β-actin was used to normalize between samples.

C - D: Total splenocytes, splenic B cell, T1T2, FO and MZ B cell numbers from cKO (HO), Het and WT mice. Data are representative of 6 independent experiments for splenocytes and B cells and 4 independent experiments for T1T2, FO and MZ B cells..

E: Splenic B cells from cKO (HO), Het and WT mice were cultured ex vivo at 37 °C with or without BAFF (200ng/ml) for the indicated times. Viability was determined by propidium iodide staining and flow cytometry. Error bars represent the standard error of the mean between experiments and data are representative of three independent experiments.

Supp. Fig. 2

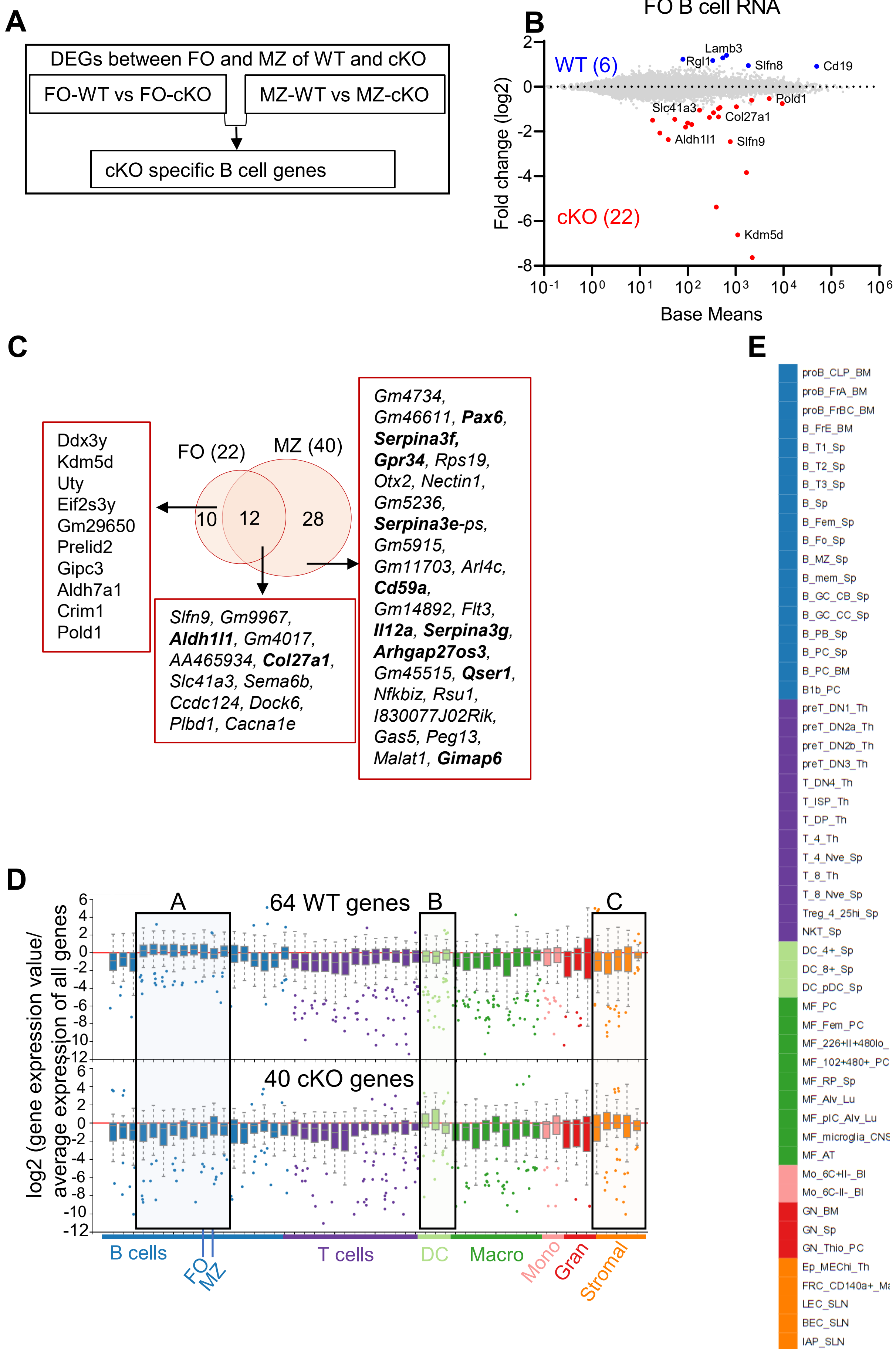
A
DEGs between FO and MZ of WT and cKO
FO-WT vs FO-cKO
MZ-WT vs MZ-cKO
cKO specific B cell genes
B
FO B cell RNA
WT (6)
cKO (22)
Lamb3
Rgl1
Slfn8
Cd19
Pold1
Slc41a3
Col27a1
Aldh1l1
Slfn9
Kdm5d
Fold change (log2)
Base Means
C
FO (22)
MZ (40)
10
12
28
Ddx3y
Kdm5d
Uty
Eif2s3y
Gm29650
Prelid2
Gipc3
Aldh7a1
Crim1
Pold1
Slfn9, Gm9967, Aldh1l1, Gm4017, AA465934, Col27a1, Slc41a3, Sema6b, Ccdc124, Dock6, Plbd1, Cacna1e
Gm4734, Gm46611, Pax6, Serpina3f, Gpr34, Rps19, Otx2, Nectin1, Gm5236, Serpina3e-ps, Gm5915, Gm11703, Arl4c, Cd59a, Gm14892, Flt3, Il12a, Serpina3g, Arhgap27os3, Gm45515, Qser1, Nfkbiz, Rsu1, I830077J02Rik, Gas5, Peg13, Malat1, Gimap6
D
64 WT genes
40 cKO genes
A
B
C
log2 (gene expression value/ average expression of all genes
B cells
FO
MZ
T cells
DC
Macro
Mono
Gran
Stromal
E
proB_CLP_BM
proB_FrA_BM
proB_FrBC_BM
B_FrE_BM
B_T1_Sp
B_T2_Sp
B_T3_Sp
B_Sp
B_Fem_Sp
B_Fo_Sp
B_MZ_Sp
B_mem_Sp
B_GC_CB_Sp
B_GC_CC_Sp
B_PB_Sp
B_PC_Sp
B_PC_BM
B1b_PC
preT_DN1_Th
preT_DN2a_Th
preT_DN2b_Th
preT_DN3_Th
T_DN4_Th
T_ISP_Th
T_DP_Th
T_4_Th
T_4_Nve_Sp
T_8_Th
T_8_Nve_Sp
Treg_4_25hi_Sp
NKT_Sp
DC_4+_Sp
DC_8+_Sp
DC_pDC_Sp
MF_PC
MF_Fem_PC
MF_226+II+480lo_
MF_102+480+_PC
MF_RP_Sp
MF_Alv_Lu
MF_pIC_Alv_Lu
MF_microglia_CNS
MF_AT
Mo_6C+II-_Bl
Mo_6C-II-_Bl
GN_BM
GN_Sp
GN_Thio_PC
Ep_MEChi_Th
FRC_CD140a+_Ma
LEC_SLN
BEC_SLN
IAP_SLN

Supp. Fig. 2:

A- Scheme demonstrating the comparison profile for the identification of differentially expressed genes (DEGs). FO genes in the WT were compared to the FO genes in the cKO, similarly WT MZ genes were compared to the cKO MZ genes.

B- MA plot displaying the log fold-change compared with mean expression and showing the DEGs in FO B cells of the WT (6 genes) and of the cKO mice (22 genes).

C- Venn diagram showing the overlapping of the 40 cKO MZ genes with the 22 cKO FO genes results in 12 genes that are *Gtf2i* cKO B cells specific. A list of the 12 *Gtf2i* cKO unique genes, the 10 cKO FO and the 28 cKO MZ unique genes are shown.

D- Box and whiskers plot shows the WT 64 MZ genes and the cKO 40 MZ genes and their expression levels in all the immune cells using ImmGen-database. Genes that are enriched in B cell compartments are shown in box A, genes that are enriched in DC are shown in box B while the genes that are enriched in granulocytes and stromal cells are shown in box C.

E- Scheme showing the cell populations as presented by ImmGen-Database and shown in all the heatmaps that are created from ImmGen-Database in 2B, 2C and Supp. Fig 2D.

Supp. Fig. 3:

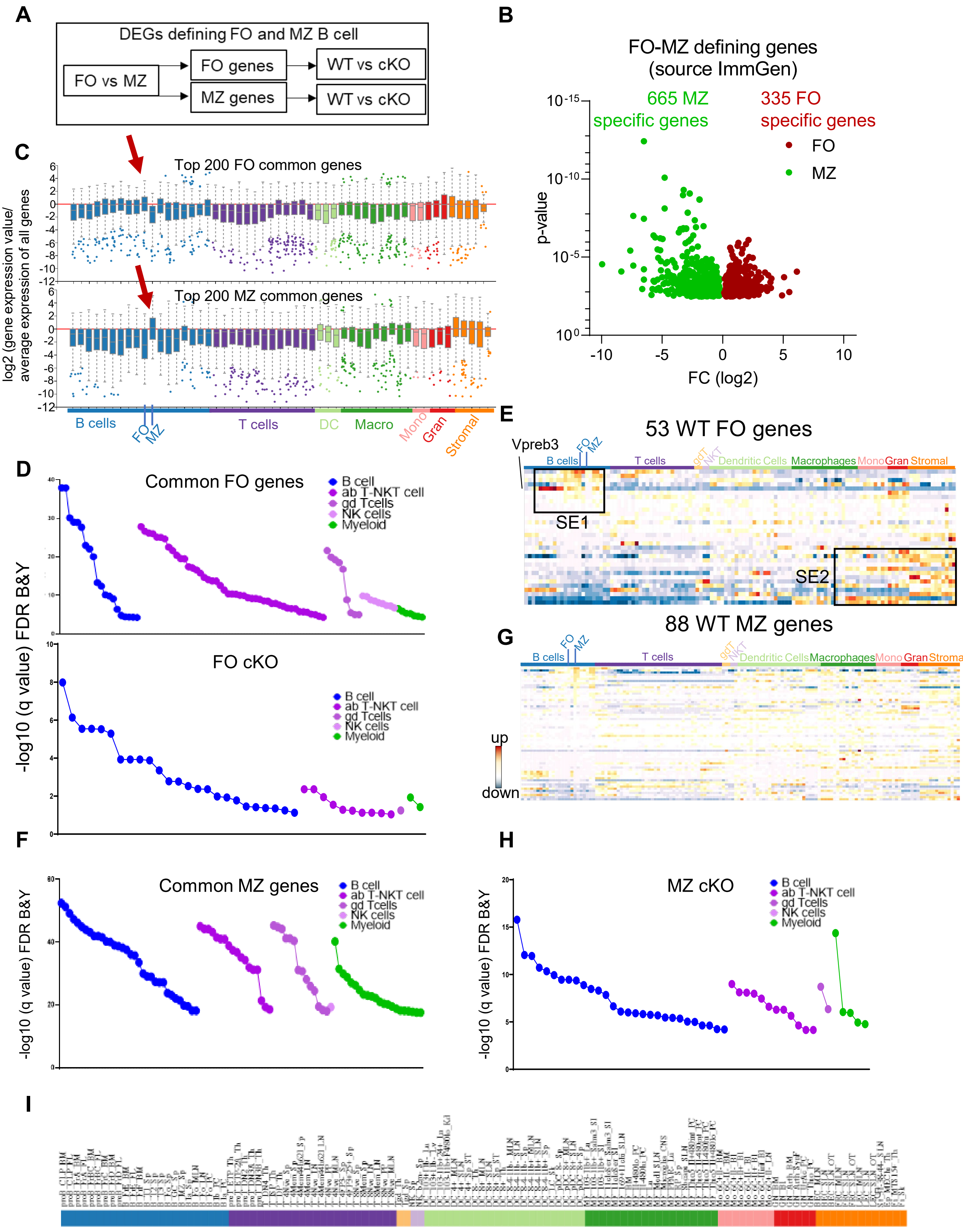
A
DEGs defining FO and MZ B cell
FO vs MZ
FO genes
MZ genes
WT vs cKO
WT vs cKO
B
FO-MZ defining genes
(source ImmGen)
665 MZ
specific genes
335 FO
specific genes
FO
MZ
p-value
FC (log2)
C
Top 200 FO common genes
Top 200 MZ common genes
log2 (gene expression value/
average expression of all genes
B cells
FO
MZ
T cells
DC
Macro
Mono
Gran
Stromal
D
Common FO genes
B cell
ab T-NKT cell
gd Tcells
NK cells
Myeloid
FO cKO
-log10 (q value) FDR B&Y
E
53 WT FO genes
Vpreb3
T cells
gdT
NKT
Dendritic Cells
Macrophages
Mono
Gran
Stromal
SE1
SE2
G
88 WT MZ genes
up
down
F
Common MZ genes
H
MZ cKO
I

Supp. Fig. 3:

A: Scheme demonstrating the comparison profile for the identification of differentially expressed genes (DEGs) between FO and MZ genes in the WT and cKO mice. First FO genes were compared to the MZ genes of the WT mice and similarly FO genes were compared to the MZ genes of the cKO mice. Second the WT FO genes were compared to the cKO FO genes, similarly WT MZ genes were compared to the cKO MZ genes.

B: Volcano plot showing the FO vs MZ genes of C57BL/6 (WT) mice from the Immunological Genome Database (ImmGen-Database). Data shows 335 FO specific genes versus 665 MZ specific genes.

C: Box and whiskers plot shows the top 200 common FO genes (common in WT and cKO) and their expression levels in all the immune cells using ImmGen-database. Genes that are enriched in B cell compartments are shown in box SD1, genes that are enriched in T cell compartments are shown in box SD2 while the genes that are enriched in monocytes, granulocytes and stromal cells are shown in box SD3.

D, F and H: Point plots showing the significantly enriched genes in the different populations of the various immune cells that was identified in the ImmGen-database as seen in Fig. 3E – F and Fig. 4C – D. According to these significant genes the boxes were plotted in Fig. 3E – F and Fig. 4C – D.

E: Heatmap showing the expression levels of the 53 WT specific FO genes in the different immune cells when they were input in the ImmGen-Database. Box SE1 shows the expression levels of these genes in the different subsets of B cells. Box SE2 shows their expression levels in the monocytes, granulocytes and stromal cells.

G: Heatmap like in E but for the 88 WT MZ genes.

I: Scheme showing the cell populations as presented by ImmGen-Database and shown in all the heatmaps that are created from ImmGen-Database (Fig. 3E - F, Fig. 4C – D and Supp Fig. 3E – 3G).

Supp. Fig. 4:

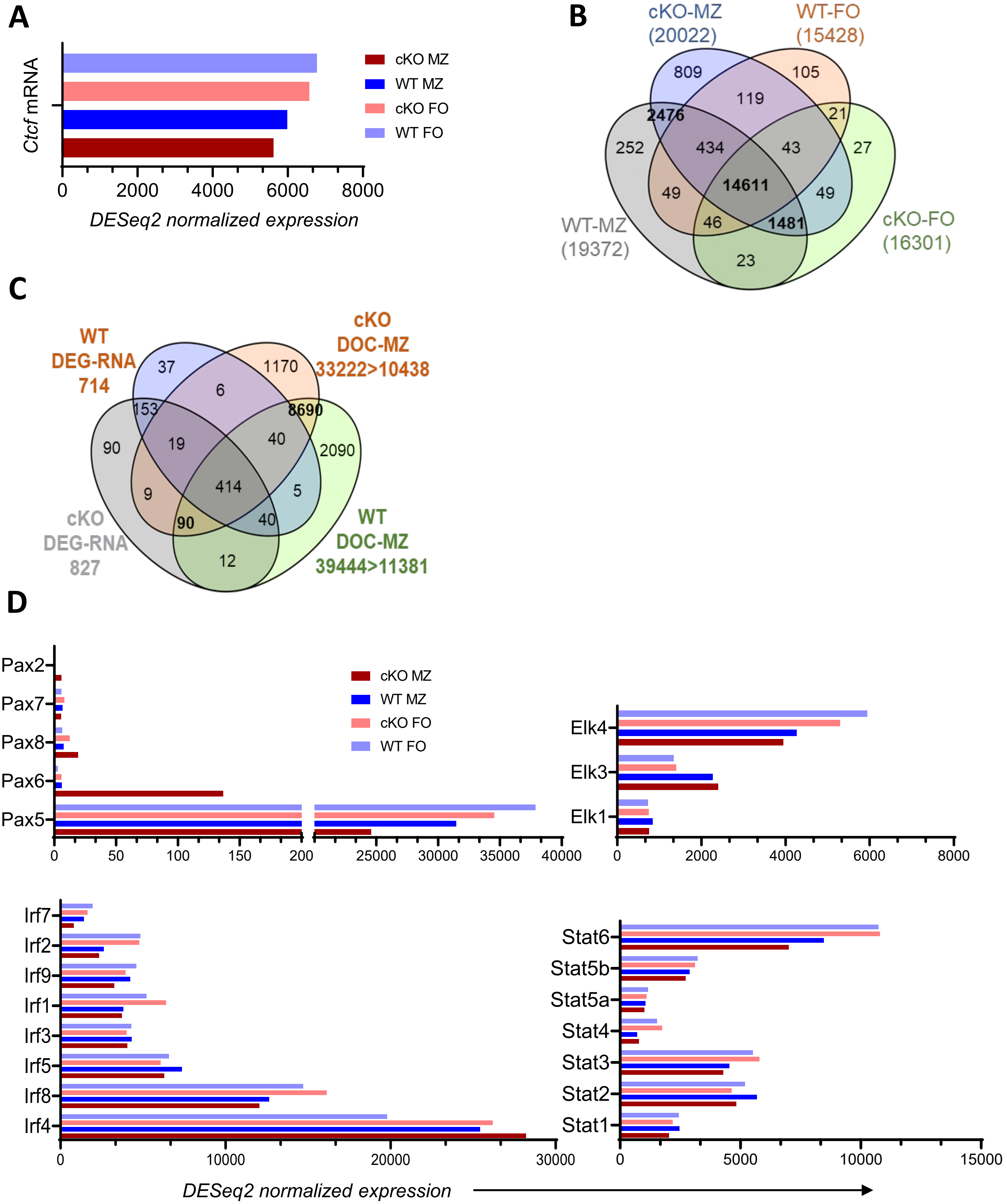
A
Ctcf mRNA
cKO MZ
WT MZ
cKO FO
WT FO
0
2000
4000
6000
8000
DESeq2 normalized expression
B
cKO-MZ (20022)
WT-FO (15428)
WT-MZ (19372)
cKO-FO (16301)
809
105
119
2476
21
252
434
43
27
14611
49
49
46
1481
23
C
WT DEG-RNA 714
cKO DOC-MZ 33222>10438
cKO DEG-RNA 827
WT DOC-MZ 39444>11381
37
1170
6
153
8690
90
19
40
2090
414
9
5
90
40
12
D
Pax2
Pax7
Pax8
Pax6
Pax5
cKO MZ
WT MZ
cKO FO
WT FO
0
50
100
150
200
25000
30000
35000
40000
Elk4
Elk3
Elk1
0
2000
4000
6000
8000
Irf7
Irf2
Irf9
Irf1
Irf3
Irf5
Irf8
Irf4
0
10000
20000
30000
Stat6
Stat5b
Stat5a
Stat4
Stat3
Stat2
Stat1
0
5000
10000
15000
DESeq2 normalized expression

Supp. Fig. 4:

A: *Ctcf* mRNA expression levels of MZ and FO B cells of WT and cKO mice.

B: Open chromatin regions as in Fig.5A were annotated to its associated genes using annotated.pl algorithm in HOMER. These genes were further compared using 4-way Venn to identify shared and unique genes for indicated B cell subsets from WT and cKO B cells.

C: Differentially open chromatin of MZ B cells as in Fig. 6A were annotated to their associated genes (as in Supp Fig. 4B) for WT and cKO B cells. These annotated genes were then overlapped with the MZ DEGs that identified from the RNA-seq data and shown in Fig. 3A-B.

D: DESeq2 normalized mRNA expression levels of the different transcription factor family members.

Supp Table 1				
**Gene**	**WT FO**	**cKO FO**	**WT MZ**	**cKO MZ**
Ryr2	382.01	308.66	26.11	20.74
Rgl1	840.01	346.27	92.18	51.68
Ngfr	304.56	181.56	28.20	12.13
Slc4a11	385.75	337.21	42.33	23.27
Cacna1i	10115.56	9013.73	1325.77	736.09
Lifr	238.28	180.59	21.29	10.62
Crisp3	467.41	425.19	60.14	32.03
Gm11346	1004.75	939.90	134.31	135.64
Gm11690	241.05	234.51	22.99	12.13
Prss12	173.72	346.21	15.88	34.11
Gm39383	119.40	60.98	7.45	5.52
Rflnb	1504.49	1363.37	262.48	256.42
Slco5a1	159.74	107.47	16.56	11.67
Crip3	188.94	160.24	24.49	26.83
Gm19951	2265.94	1999.93	414.43	338.56
Cdkl1	290.00	253.07	47.99	36.55
Gad1	496.18	280.77	77.98	28.03
Mapk12	768.62	676.85	127.95	99.40
Col27a1	402.53	1097.73	71.89	183.64
Dmwd	423.58	267.75	75.15	33.94
Fry	3008.63	2558.38	593.10	469.09
Ndrg1	207.07	253.46	27.10	19.78
Dusp3	617.27	546.83	106.40	75.51
Wfdc17	127.89	163.77	15.26	18.63
Mapk11	4319.14	4139.79	910.05	748.53
Fam102b	617.18	592.88	121.46	129.71
Loxhd1	69.04	41.40	5.12	2.54
Hcrtr2	192.74	189.37	23.41	13.13
Vim	9654.25	9311.42	1943.21	1442.64
Dmpk	268.23	148.22	46.40	28.51
Trib2	2570.02	3031.38	537.25	583.53
L1cam	652.07	645.44	136.79	108.97
Alcam	977.35	813.09	207.07	147.38
Lamp3	179.85	165.78	31.77	42.17
Fcgrt	976.48	930.94	203.47	170.61
Fcer2a	30480.27	37351.30	4740.69	6476.49
Cd55	20241.06	22592.55	4685.17	5972.21
Lrrc23	69.77	69.27	7.39	3.56
Itga6	936.41	1058.55	194.52	176.75
Kcnb1	540.18	485.23	111.60	103.97
Iqcn	134.84	117.90	7.53	8.68
Prkcg	145.87	125.84	24.94	14.68
Gpr146	935.40	809.83	209.70	173.90
Ttbk1	435.81	744.71	92.42	133.63

Gadd45g	908.43	1074.66	193.68	270.50
Gm45552	784.76	673.40	168.45	140.88
Sorcs2	185.04	118.01	29.50	18.65
Sdc1	361.64	426.89	63.75	45.01
Ccdc114	178.82	153.75	36.81	38.10
Itgb7	7168.33	7242.77	1818.80	1592.37
Bach2	9186.68	8906.56	2369.30	2356.71
Rbpms	228.89	369.06	50.31	99.18
Nacad	340.94	272.76	76.44	49.75
Stac2	1221.96	1119.25	307.25	274.65
Gm15675	4079.17	3707.58	994.90	965.87
Bcl6	3553.62	3800.44	925.68	787.26
Atp1b1	429.02	297.60	96.57	58.45
Maf	274.64	464.16	53.04	88.01
Ahnak	7515.09	5912.97	1224.37	472.23
Ssbp2	270.06	381.56	59.85	91.13
Gm34225	96.28	124.91	14.89	18.30
Pgap1	9030.20	9151.93	2519.39	2309.79
Emp3	2447.44	2217.07	652.46	486.36
Gm8369	4834.54	5151.18	1308.75	1220.55
Gmfg	3794.03	4459.24	1056.94	1423.81
Qrfp	137.97	137.42	29.07	24.37
Slc2a6	564.91	627.62	148.01	155.87
Enpp1	1385.59	1226.09	392.38	336.59
Myadm	1530.87	1164.66	202.76	94.33
Gm37352	377.77	341.52	91.15	60.10
Cd55os	1530.67	1306.68	403.45	357.91
Scimp	438.61	393.64	112.62	91.44
Crybg3	1307.38	1234.90	362.17	313.77
Tmprss13	60.13	61.32	8.78	3.06
Zfp318	16463.98	15951.14	5009.44	4303.39
Ldlrad4	88.58	113.41	19.08	22.27
Dll1	157.05	199.35	37.77	32.07
Rnf144a	4265.08	3896.73	1301.11	979.23
Mex3b	229.34	209.42	62.26	56.32
Tctn1	1602.98	1487.08	506.12	431.26
Kcnj1	65.44	73.12	6.88	8.08
Lef1	106.14	120.46	15.99	12.13
Csrp2	263.09	238.86	63.95	41.36
Mturn	424.20	357.14	129.99	132.22
Gm8251	155.11	99.61	30.77	18.18
Gm43196	192.12	194.87	54.53	61.25
B3gnt5	11640.16	12188.82	3823.36	3806.76
4632428C04R	192.27	172.37	44.87	37.27
Gm16214	173.44	157.72	48.93	42.07
Cdc25b	3949.16	4005.77	1266.81	1171.79
Rassf3	4198.63	4447.44	1377.03	1323.84

Ldlr	1235.35	1128.55	358.82	306.09
Cdkn2d	928.71	868.49	276.31	247.34
Vmn2r97	396.61	330.29	120.08	86.66
Arhgef18	12799.17	12138.40	4226.16	3849.49
Cd200	1501.94	1824.45	480.64	476.65
Gm43769	48.26	73.86	7.87	14.78
Cdkn1a	85.54	73.69	16.73	14.13
Pygm	225.26	177.93	68.55	61.92
Lfng	1877.89	2027.88	631.96	638.76
Cpm	2923.84	2742.41	971.87	823.46
Trim59	2273.05	2392.27	725.48	865.84
Arhgef3	7978.86	8223.56	2685.47	2830.30
Tent5a	1418.33	1033.94	474.54	316.10
Igf1r	98.85	171.33	25.90	27.38
Tle1	473.12	508.01	151.01	131.33
B3gnt7	607.96	701.21	174.69	131.28
Gm23037	65.51	70.83	14.53	13.71
Bach2os	126.93	144.82	32.47	29.85
Smad1	233.65	124.90	68.66	43.98
Tagap	2551.37	2765.03	885.71	941.67
Ighv1-5	197.21	267.37	56.99	90.53
Cmah	10453.48	8161.25	3495.26	2827.21
Rgcc	163.47	201.44	25.66	22.91
Adap1	673.14	759.20	234.37	246.06
Il4ra	12800.42	9503.37	4678.31	3343.54
Bmf	2061.03	1561.88	748.24	450.63
Neurl3	9284.40	9114.96	3358.23	3437.41
Gm5511	118.98	123.87	32.95	44.32
Rhebl1	523.10	513.23	183.28	232.70
Trim46	73.25	38.66	15.17	6.56
Stard8	76.56	123.65	17.21	40.32
Orai2	2617.79	2358.41	962.01	920.95
Sell	30972.08	29597.09	11266.44	13080.35
S100a10	3308.44	3584.51	1172.27	1054.89
Diras2	172.35	205.85	55.12	53.77
Fchsd2	13047.43	13740.93	4946.70	5053.01
Tnik	568.34	707.28	203.51	240.32
Sele	61.84	59.25	11.55	7.54
Kctd14	468.29	289.32	69.63	28.82
Spns2	2178.00	1866.28	793.72	561.73
Pde4c	121.68	105.03	33.69	22.80
Scn8a	85.65	82.36	20.78	13.80
1700001K23F	99.23	94.62	25.37	25.28
Aida	2342.06	2201.24	886.41	831.61
Itih5	452.24	321.00	152.61	109.06
Sfxn3	1347.92	1274.14	501.72	509.15
Serinc5	1442.83	1181.38	513.71	316.62

Pde2a	29.86	61.11	3.28	5.02
Sbk1	12795.27	11593.17	4975.36	3397.69
4932442E05R	206.84	222.80	67.86	66.99
Ccr7	15259.10	15424.40	4507.61	3558.71
Gm26532	552.67	523.59	89.61	64.93
Gm36931	80.55	90.71	19.17	17.77
Cbx7	11932.12	11416.15	4692.87	4693.15
Zfp608	4103.13	4348.79	1577.44	1387.29
Lmo2	7104.82	6372.44	2747.05	2832.89
Nectin1	115.48	268.49	35.51	114.57
Hvcn1	9139.81	8864.88	3548.76	2684.06
Selp	36.13	58.52	6.94	15.21
Sgpp2	146.72	126.47	46.58	33.82
Gimap3	10173.31	9331.27	4035.91	3955.27
Gm8995	18953.59	17197.63	7505.14	7116.62
S1pr4	3037.43	2668.09	1103.95	822.93
Ifnlr1	122.89	156.04	40.80	29.39
4930511E03R	43.94	21.99	5.94	0.52
Sorl1	22706.14	21079.89	9299.09	7777.04
Trim16	27.10	30.04	2.77	5.04
Cpne5	116.17	104.07	33.72	18.65
Ank	416.05	461.12	162.44	192.97
Gm19261	134.43	109.50	42.21	33.84
Pgpep1l	54.88	71.07	11.49	12.65
Gm48038	52.17	40.51	11.58	10.65
Dyrk2	2409.17	2324.78	974.27	953.95
Slc4a3	57.99	39.50	13.45	6.12
Adamts6	1758.92	1425.40	700.58	636.33
Gm35065	20.13	30.11	0.00	1.51
Rasa3	14193.39	13790.57	5803.38	5125.13
Cisd3	379.73	375.19	145.50	150.20
Gm19585	349.45	300.33	136.33	120.57
Gm15880	104.33	120.96	32.37	36.71
Maml3	431.45	526.83	168.13	195.76
Ccdc180	83.25	124.65	26.98	44.63
Fam107b	16520.55	16431.24	5532.99	4934.32
Sfn	944.40	1084.12	378.96	408.13
Abca1	6645.63	6769.47	2727.43	2387.11
Extl1	272.10	244.19	103.74	94.67
Cd93	530.55	579.98	175.02	128.94
Abca6	50.35	61.44	13.05	18.18
Ighd	57427.08	63616.26	16195.54	18339.50
Capn5	886.66	743.61	354.61	323.30
Zc3hav1l	225.98	264.27	85.54	59.44
Itgb3	1242.46	1137.99	505.89	498.13
Bbs9	728.14	864.80	302.48	393.83
Gm15987	1193.93	1208.25	491.85	495.17

H3f3a	2553.24	2423.31	1077.67	990.33
Antxr2	740.94	702.19	302.72	260.42
Satb1	7112.76	7298.97	2855.16	2336.45
Cc2d2b	86.09	98.74	25.57	31.26
Cldn10	101.27	86.56	31.85	32.27
Slc26a10	465.01	522.24	190.47	204.23
Amn	73.07	65.41	20.73	16.14
Tgfb3	161.89	139.89	56.98	43.29
Lmna	163.52	165.87	39.50	39.01
Ddah2	119.86	115.48	43.09	42.98
Dnah8	791.94	554.12	191.98	95.31
Mybpc2	334.82	324.38	134.17	101.46
Sh3bp5	9024.40	8759.53	3936.97	3460.45
2810405F17R	55.57	58.42	13.90	15.69
Tcp11l2	4973.60	4577.67	2176.46	1865.99
Trib3	53.53	58.19	13.68	13.20
Il6ra	1365.01	1710.33	572.86	546.10
Gm16589	173.71	148.60	67.81	62.31
Lbh	14264.22	12824.69	6289.63	5299.52
Add1	10321.79	10171.45	4560.59	4258.46
Xylt1	3613.00	3349.66	1583.81	1273.53
F2rl1	30.03	52.14	3.68	9.23
Tnnt3	156.23	143.16	59.43	47.60
Tuba1a	497.35	540.53	202.81	195.38
Slc52a3	78.66	59.04	25.80	14.69
H1f2	1208.65	1701.01	521.19	608.99
Fli1	4792.99	4265.21	2100.33	1722.23
Akap12	1096.65	811.12	435.60	303.56
Nab2	1088.96	814.34	473.29	252.98
Ripor2	15052.86	13704.05	6651.48	5225.49
Arhgap31	237.80	233.05	75.15	53.69
Slc15a3	480.79	370.59	192.71	130.28
Nsmce1	1338.66	1254.71	572.71	510.52
Stum	50.38	79.23	15.37	12.15
Flna	31897.05	28359.81	14432.30	11688.91
Scml4	2952.80	2757.85	1306.91	1319.98
Igsf9	293.70	351.05	115.28	101.38
Ssh2	11598.44	11676.21	5060.13	4681.70
Samhd1	19752.04	19496.53	8887.15	8556.76
Vsir	702.01	634.33	289.83	233.99
Gm6421	2151.83	2005.62	973.91	855.15
Eps8	286.80	306.47	116.13	98.49
Gm47782	20.76	33.18	1.87	2.51
Galc	88.79	90.58	31.45	12.73
Baiap2	580.89	582.09	256.38	157.19
Prr7	369.42	364.37	152.51	154.70
Otud1	2058.83	1969.37	925.77	849.24

Dipk1a	1840.54	1909.31	840.97	732.90
Zfp53	1419.54	1727.64	621.90	733.36
Pdp1	462.78	487.88	199.66	204.37
Il24	61.43	98.81	21.91	27.84
Marveld2	811.13	812.47	365.15	350.24
Serp2	48.45	62.65	13.42	9.59
E430014B02R	214.22	192.68	69.66	57.28
Ehd4	2004.89	1856.53	900.80	766.05
Add3	13119.63	10806.01	5829.38	4493.98
Klf3	5709.44	5050.24	2653.50	2129.19
Dgka	10159.94	9721.23	4760.68	4572.02
Tmem64	2776.86	2436.13	1286.48	970.43
Tub	47.21	45.60	14.38	7.65
Rhoh	4727.60	4908.45	2215.80	2246.12
Kif21b	21167.74	18876.36	9978.82	8196.54
Fam221a	71.72	97.80	26.44	21.26
Tnfrsf12a	206.77	216.94	84.50	67.59
Lilra6	239.31	158.71	95.57	51.64
Btnl2	625.75	649.41	290.56	295.67
Retreg1	4461.72	4374.48	2112.89	2037.41
Clcf1	5467.09	4659.27	2577.11	2012.42
Treml2	7706.86	7176.45	3672.88	3486.40
Fsip1	52.54	62.99	16.34	12.22
Fam160a1	60.16	81.13	21.38	25.31
Gm11342	56.68	43.52	16.08	8.08
Fkbp11	93.11	89.41	34.65	17.67
Gm9706	58.49	64.35	16.91	17.12
Pgm2l1	980.48	857.64	452.92	357.98
Ptk6	42.17	42.80	12.23	10.12
Grap2	8423.23	7495.13	3903.66	3082.18
Gucd1	3695.05	3420.15	1770.65	1405.04
Apbb1	75.14	88.50	27.04	33.63
Rasl10a	31.24	29.04	7.45	1.55
Cerk	9869.19	9519.30	4772.85	4067.63
Stat4	1527.20	1744.75	700.60	780.77
Cgas	75.99	53.61	25.06	15.16
St6gal1	8622.24	8915.17	4045.53	3708.63
Btla	19098.77	23680.38	8868.69	10787.20
Rph3al	301.18	285.03	137.61	121.95
Rfx2	121.42	159.91	48.24	37.31
Lmbrd1	5613.78	5518.59	2723.56	2374.01
Gm47232	74.29	75.32	26.75	21.79
Begain	165.90	160.58	74.30	68.41

Supp Table 2				
**Gene**	**WT FO**	**cKO FO**	**WT MZ**	**cKO MZ**
Zfp36l2	16631.20	11978.28	8539.57	3427.26
Gm28935	30.81	48.12	9.85	5.10
Gm36955	55.29	73.24	21.91	12.73
Dipk2a	262.13	249.76	136.86	70.49
Eya1	196.79	125.90	97.40	31.17
Klf10	456.02	527.72	223.88	173.76
Gfi1	354.93	460.66	197.89	154.53
Ighv14-4	195.35	213.00	95.09	65.18
Rtl5	70.01	74.36	29.81	20.69
Igkv8-21	355.55	287.54	176.66	107.05
Vat1	478.15	493.71	235.43	188.01
Dusp10	580.78	509.23	328.44	172.49
Calcrl	451.19	437.02	241.28	161.06
Thbd	43.67	55.84	25.14	14.18
Arc	72.26	83.04	36.38	24.88
9030404E10Rik	67.12	58.85	22.56	11.07
Cd209a	31.43	32.06	8.72	1.99
Cacna1d	333.31	275.55	170.65	104.71
Gabrb1	19.23	42.98	9.46	6.12
Slc26a11	1076.18	1173.84	527.20	479.30
Il21r	8104.14	6061.95	4006.79	2557.93
Hlx	73.83	90.77	37.89	28.34
Plpp2	128.31	116.94	68.49	40.23
Dusp4	1346.33	2116.74	364.24	412.42
Spred2	199.39	189.62	86.09	63.98
Ighv8-6	17.38	33.62	5.58	4.61
Orm2	70.94	75.66	27.49	18.22
Pold1	5565.73	8136.02	2812.52	3480.47
Trim25	11110.55	9058.02	5359.01	3705.25
Rnf125	86.38	85.81	20.51	5.57
Gm49105	598.65	599.97	294.73	248.00
Lpp	2315.21	1914.09	1107.71	793.18
Gm50107	117.74	127.25	59.97	44.51
Igkv4-61	48.73	58.92	38.08	17.70
Shisa8	167.09	199.09	93.96	64.02
Rap1b	12257.71	12415.47	5906.56	5272.09
Rasa4	710.64	933.86	349.39	401.89
Kcnh4	89.04	89.13	37.29	25.09
Acsl3	591.95	617.32	294.65	263.11
Papln	24.60	36.48	12.74	5.62
Trip10	285.28	259.00	134.16	106.10
Gm48591	25.69	36.21	6.48	4.09

B4galnt2	53.85	50.14	19.03	13.63
Tagln2	3174.17	3300.99	1563.42	1386.46
Afap1	210.34	259.05	114.15	104.57
Ckap4	946.25	852.45	467.13	364.96
A3galt2	81.74	81.86	33.49	27.86
Aff3	5203.11	5051.79	2733.25	2216.78
Cd2	5854.94	5614.87	3251.40	2493.45
Rps6ka5	1722.17	1892.57	917.18	838.04
Brpf3	2603.32	2599.54	1279.85	1152.22
Ms4a4c	4730.68	4927.82	2079.12	1672.50
BC049352	302.42	310.29	148.30	112.63
Gm20559	4848.04	4536.93	2387.95	2009.14
Gpd1l	6990.70	7611.97	3482.68	3440.69
Gfod1	539.39	413.34	165.82	63.09
Gna15	406.48	490.42	214.76	212.93
Fxyd5	1887.35	2004.69	935.22	903.99
Cd83	11691.11	13676.48	6360.98	4985.05
Tbx6	384.80	327.44	182.20	141.18
Mtss1	6334.50	5628.23	3526.00	2582.06
Pisd-ps1	1768.54	1533.28	1075.34	694.56
Hspa4l	838.67	892.71	395.88	248.72
Mef2c	15906.81	14224.79	8123.50	6559.91
Gm35019	2.47	30.12	0.93	0.50
Mfsd6	317.02	380.75	179.09	169.50
Nav2	39.53	69.27	24.78	24.33
Tspan18	13.33	30.16	4.67	4.55
Rictor	4537.48	4355.04	2354.05	2000.15
Ier5l	126.70	132.55	57.25	51.44
Ms4a4a	75.11	78.44	28.89	25.28
Ccm2	5983.75	5235.64	3086.45	2440.59
Chrna2	209.93	245.91	92.12	99.14
Cyp2d22	250.69	274.60	119.29	120.80
Tec	4446.37	4395.25	2436.90	2006.21
Lrrc8a	284.95	328.56	139.30	146.80
Cdkl5	156.90	142.69	90.03	58.36
Fer1l5	193.52	255.48	125.93	94.38
Prr18	24.08	32.84	5.55	7.11
Cecr2	1019.59	826.47	531.84	347.00
Rab6b	238.36	275.65	116.71	121.04
Gm7707	13.36	24.68	18.37	1.01
Plekho1	5253.39	5261.01	2793.89	2493.16
Chst15	3582.53	3609.57	1989.28	1700.80
A430088P11Rik	39.25	61.48	16.22	20.23
Tnks1bp1	1280.63	1225.27	721.26	572.03

Fgd3	6465.39	6115.49	3474.99	2872.35
Ndnf	30.62	34.29	9.34	8.13
Gm37176	130.20	113.66	56.91	44.53
Atp6v0a1	3228.93	3367.74	1757.62	1524.73
Gm20627	17.59	30.83	5.58	6.53
Tmem65	305.12	328.50	169.68	149.92
Klra1	31.93	28.03	7.36	5.04
Lsp1	18122.40	17906.91	9879.47	8669.80
Gm30948	15.86	23.39	0.45	1.50
Gm37593	94.52	91.27	38.56	35.49
Rgs13	64.79	74.40	24.23	18.35
Ighv1-36	42.20	71.72	28.74	24.20
Cnn3	3528.57	3435.14	1754.89	1651.51
Kif19a	206.10	171.93	103.15	76.96
Reck	26.14	34.69	8.86	6.15
Rara	2533.56	2599.46	1277.52	1256.60
Dennd5b	4248.45	3444.06	2093.19	1634.71
Cplx2	68.80	91.80	44.02	33.55
Lasp1	2955.84	2805.01	1581.89	1378.37

Supp Table 3				
**Gene**	**WT FO**	**cKO FO**	**WT MZ**	**cKO MZ**
I830077J02Rik	56.87	44.20	3562.91	5070.75
Asb2	47.84	33.45	2503.44	2433.18
Gpr156	10.18	2.18	772.65	781.21
Ackr3	43.72	42.92	2501.07	3113.03
Adam28	9.79	5.20	668.52	652.74
Tsga13	11.72	7.94	794.39	869.39
S1pr3	241.93	198.18	7570.85	8085.39
Nebl	93.26	111.17	2850.30	3608.07
Plxnd1	235.96	233.44	6288.00	7490.84
Rgs10	55.42	47.89	1515.88	1277.12
Tbc1d9	89.31	150.56	2358.74	3373.35
Rxfp1	11.72	9.06	481.11	393.37
Pla2g7	26.13	25.51	830.30	801.95
Ceacam2	14.10	13.66	590.13	640.31
Rap1gap	87.64	72.57	2224.96	1517.64
Ackr2	53.59	52.82	1678.37	2102.60
Ffar2	53.42	55.04	1374.25	1656.27
Gm2065	8.77	7.05	350.09	312.03
Rec8	18.11	35.93	610.50	629.55
Trpm2	54.44	59.37	1337.95	1674.55
Nrp2	31.32	29.77	835.11	1080.36
Myof	613.99	579.07	11868.65	9884.74
Zfp532	2.92	5.49	271.65	313.01
Tmod2	5.26	3.25	262.34	188.38
Serpine2	9.82	10.91	407.98	372.59
Cd9	92.97	79.56	1582.65	1452.13
Srgap3	30.43	12.53	711.95	406.48
Zc3h12c	277.18	295.04	4118.69	3548.02
Grip2	7.75	11.42	314.33	309.40
Ms4a14	3.83	7.00	209.19	136.30
Sema7a	506.98	440.76	7191.19	4766.05
Sorbs2	19.49	18.24	456.55	392.54
Il10	11.72	15.50	282.12	265.34
AW011738	159.08	124.84	2149.72	2184.74
Ube2e2	0.97	1.12	135.92	200.21
Gm4956	6.32	6.94	182.40	148.22
Lipc	30.53	16.06	543.74	361.78
Ms4a7	8.32	10.52	263.63	237.28
Myo18b	1.93	1.12	139.09	162.07
Ldlrad3	78.67	82.08	1076.94	909.77
Fcrl5	516.84	463.80	6390.63	6834.72
Cd300lf	132.38	116.09	1762.66	1726.80

Grk3	180.44	201.08	2070.98	2077.80
Atxn1	212.61	219.89	2352.96	2181.03
Lair1	46.19	43.92	530.38	728.27
Cdh17	209.35	201.76	2105.17	2347.27
Tlr3	131.59	117.16	1294.18	1072.16
Smyd2	13.17	23.28	214.97	296.62
Rundc3b	24.55	13.32	374.43	343.98
Cdc42bpb	140.82	192.82	1434.17	1560.33
Alpl	59.23	56.12	737.68	721.79
Ptpn14	533.26	327.41	4907.51	4095.29
Csf2rb	321.22	279.89	3036.63	2601.74
Pde4a	348.69	338.19	3011.62	1962.51
Cd36	749.78	635.11	5785.48	4965.26
Dse	40.01	27.87	377.65	197.82
Mfhas1	664.40	481.31	4845.20	3412.53
1700071M16Rik	1.00	2.18	72.34	61.52
Stc1	1.00	2.18	65.15	108.16
Gm43916	5.33	7.22	177.02	132.38
Gm16793	2.47	2.13	87.06	66.90
Tspan15	25.60	11.92	1939.25	1589.32
9630028I04Rik	41.27	45.93	374.13	425.90
Tnfrsf21	80.51	97.14	678.53	631.87
Nlrp1b	8.75	13.82	118.63	171.92
Zfyve28	59.53	52.31	484.12	490.53
Ptafr	9.25	1.12	135.11	70.25
Sel1l3	8.77	4.25	123.08	56.38
Drd5	1.50	0.56	117.01	71.37
Gm15513	43.06	37.09	366.54	394.88
Lilrb4a	114.09	120.43	999.85	972.77
Nedd4	1246.76	1446.03	8128.17	8879.16
Dph5	734.99	753.75	4639.04	4882.63
Oosp1	10.20	10.01	121.15	122.98
Slit3	3.90	1.12	83.27	61.82
Dyrk4	9.34	18.35	118.62	188.64
Sema6b	35.97	116.77	289.85	691.13
Slc39a4	23.39	22.94	196.18	224.46
Cd59a	4.37	10.40	76.93	196.67
Cacnb4	24.46	10.01	212.32	97.94
Stom	115.98	101.50	761.44	662.62
Cd1d1	1034.37	1101.67	6329.27	8019.74
Pdia4	4925.05	5055.92	28704.75	28764.13
Gml	4.40	2.57	81.29	58.40
Sirpa	163.30	119.13	1139.70	924.20
5730409E04Rik	3.92	8.95	76.62	122.15

Vash2	4.37	5.88	79.13	137.89
Cyp39a1	252.33	221.09	1591.24	1292.31
Marcks	1364.13	1140.41	7620.71	6450.34
Mzb1	156.07	181.51	960.52	918.89
Dzip1	9.77	24.45	110.29	131.59
Gdpd5	15.62	22.21	140.19	133.87
Lrrc25	57.04	42.80	425.64	369.71
Gpr55	48.29	18.80	406.25	228.87
Abcb1a	39.38	20.04	900.30	587.96
Ppl	99.00	124.19	1494.17	1611.81
Igkv2-107	20.87	11.53	202.79	113.54
Coro2b	8.16	2.57	115.68	76.82
Cyp8b1	1.00	4.14	53.31	52.19
Trim30c	68.97	47.28	414.99	280.07
Galm	6.80	5.20	83.44	73.47
Cln8	22.60	16.51	230.02	119.51
Fam149a	4.92	1.12	84.30	63.26
Gm12503	7.23	6.05	98.30	85.96
Plau	7.91	12.20	196.59	172.66
Kcnk13	30.05	23.16	223.27	198.01
Dtx1	4656.89	4986.58	24720.93	25031.32
Guca2b	1.47	4.31	57.11	35.06
Trem1	31.99	45.55	233.13	336.21
Lysmd2	25.75	17.57	177.46	168.31
Hbegf	2.45	3.86	57.90	73.54
Hmgn3	134.16	107.87	708.83	645.29
Ntng2	38.78	82.96	283.48	433.84
A930005H10Rik	119.54	98.64	642.48	664.24
Dok2	33.59	41.97	218.75	210.48
Neb	36.13	16.73	224.63	178.09
5033430I15Rik	5.44	6.27	74.94	85.53
Gm42372	13.17	16.28	104.79	98.92
Gm4610	189.91	169.18	980.95	1097.10
Slc22a3	1.47	3.92	77.51	46.78
Grn	960.92	807.68	4508.69	3928.06
Tns1	18.97	8.06	153.18	96.96
Nhs	16.59	32.39	119.28	164.12
Cdon	323.82	311.49	1536.37	1723.31
Cfp	574.16	680.97	2752.76	3548.62
Grm6	72.55	85.78	371.78	348.15
Ttc8	9.79	18.91	84.89	102.96
Myo6	58.82	46.66	292.53	332.04
Gm32834	0.97	1.00	43.71	44.53
Prf1	65.79	51.87	354.58	363.90

Ryk	8.23	8.11	79.79	85.89
Bst1	22.16	19.69	169.09	299.37
Gm45867	203.99	176.81	952.85	717.40
Gm37422	8.77	6.10	91.63	127.86
Terb1	167.98	128.33	803.96	770.07
Adamdec1	9.68	10.69	81.89	96.10
Cd1d2	9.75	8.22	84.12	138.08
Agrn	202.69	164.72	918.06	892.27
Dusp16	989.28	927.01	4824.48	4271.18
Plac8	4818.04	6232.70	20282.58	20831.89
Slc22a17	44.41	59.53	230.30	277.71
Cfap126	12.10	11.58	90.21	77.01
Nod2	87.62	77.44	418.79	310.67
Cish	8.32	14.44	86.00	55.06
Meis3	121.08	124.95	587.29	482.35
Vwa5a	170.50	181.45	803.05	884.79
Mpeg1	690.13	603.02	4760.20	4544.16
Spag5	55.75	50.30	369.71	306.46
Sla2	31.62	23.67	177.05	109.07
Eppk1	42.41	31.94	208.47	188.54
Ahr	294.70	219.91	1367.48	1317.46
Epm2a	11.74	15.33	82.02	93.76
Npr2	9.72	3.81	85.67	62.59
Gm11707	22.87	33.28	136.71	139.90
Jph1	2.42	3.25	46.10	62.83
Dkkl1	19.85	21.82	127.13	146.09
Tm6sf1	2165.06	1946.66	8617.21	7431.73
Alpk2	115.52	92.94	522.96	449.41
Dtx4	177.73	200.80	729.02	713.60
Aldh5a1	3.92	3.64	49.64	62.35
Slc6a13	4.35	6.94	49.04	79.02
Akr1e1	41.20	51.70	198.79	219.13
Txlnb	4.37	3.64	53.32	39.94
Pclaf	52.95	33.40	363.88	340.02
Il9r	3692.93	4416.20	14469.04	20323.21
Rsu1	657.46	748.34	2607.60	3734.39
Ccr1	50.05	32.50	643.37	757.88
Bub1	27.44	18.75	209.18	158.43
Adamts13	0.95	3.08	38.17	54.73
Bfsp1	4.87	2.13	51.76	41.54
Gbp8	146.80	117.04	596.10	514.41
Kntc1	38.61	23.67	258.23	255.88
Espl1	39.04	26.58	282.49	208.06
Id2	220.41	185.78	909.14	699.78

Pik3r4	3356.40	2977.81	17530.46	15237.65
Chrm3	0.50	0.50	34.68	59.27
Endod1	388.94	354.20	1467.81	980.23
P4htm	39.22	47.00	185.52	156.94
Asap2	167.50	141.06	640.11	562.27
Gm5547	29.40	18.52	144.60	135.50
Rhobtb1	7.68	10.74	80.68	58.67
Camkmt	127.94	93.89	506.44	522.72
Pmepa1	77.94	52.22	400.58	287.44
Zap70	55.58	31.89	265.52	139.99
Kif18b	18.31	8.56	153.12	115.64
Slc9a9	56.37	32.62	234.32	183.61
Ly96	68.40	63.28	278.27	241.45
Rhbdf1	672.33	1015.96	2545.91	3738.96
Mybl2	60.62	53.65	257.14	282.02
Tpst2	1342.28	1653.35	5191.04	4781.05
AF067061	6.71	5.26	72.56	34.35
Emb	92.54	93.24	409.50	495.67
Plcd3	17.88	10.86	638.52	330.00
Cr2	25346.33	24148.84	88341.18	92019.62
Akap1	17.02	14.71	90.66	111.46
Hs3st3b1	89.78	85.11	356.06	295.94
Inpp4a	594.48	721.46	2130.76	1663.45
Ell3	65.08	77.55	263.53	262.78
Dglucy	239.70	280.04	862.77	963.04
Gm36936	21.16	21.65	132.57	113.63
Cdca5	28.17	29.16	165.39	162.24
Tubg2	66.31	62.38	277.99	237.95
Jaml	169.00	149.45	624.00	504.75
Lurap1	102.07	86.94	410.08	443.12
Gm22748	39.81	68.32	181.67	191.91
Epcam	83.86	113.81	344.38	289.69
Crebl2	1030.78	1038.03	3573.83	3626.24
Clspn	37.23	37.10	235.98	208.66
5830418P13Rik	72.98	47.50	281.13	295.41
Fam241b	1.00	2.57	34.66	41.09
Mlkl	7.30	9.57	56.17	51.80
Disc1	12.19	10.29	80.25	97.58
Afdn	23.44	15.05	112.41	113.63
Pde8a	335.74	364.03	1268.06	1262.01
Kifc3	13.01	14.71	80.90	77.16
Ccna2	60.42	39.17	351.11	318.52
Slc39a11	93.83	142.40	342.38	369.14
Abcb1b	97.78	83.71	363.26	328.48

E2f8	23.63	14.71	137.61	117.06
Mgst1	43.88	48.68	183.86	219.93
Cenpf	47.11	24.68	281.87	243.58
Tmtc4	144.52	158.70	566.66	578.83
Hdgfl3	243.93	230.70	846.59	883.37
Mcm10	30.39	17.74	184.55	190.15
Gm31499	1.93	1.01	38.37	43.86
4930568A12Rik	18.90	21.87	95.95	114.04
Ldhd	9.77	17.96	61.99	73.86
Derl3	101.64	109.26	427.36	440.23
Dnase1l3	125.43	73.35	1020.76	668.77
Mvb12b	17.43	24.56	91.71	100.10
Gm42047	137.69	67.53	485.45	297.68
Osgin1	102.00	152.29	441.54	517.40
Iqgap3	11.13	6.83	96.95	79.93
Gm38244	14.96	12.42	94.64	97.16
Itm2c	247.94	303.49	893.67	834.63
Perm1	90.85	82.31	322.99	368.12
Hes5	42.29	26.13	191.57	117.13
Mocos	4.40	2.07	44.23	42.50
Tpx2	64.76	40.62	308.31	247.53
Fam92a	55.89	41.01	211.95	234.16
Pde4d	235.90	271.95	2059.20	1836.40
Insrr	0.48	3.02	42.08	35.93
Ube2c	20.30	12.64	142.25	119.49
Carmil1	1074.00	1083.12	3471.79	3459.07
Rbm47	23.35	20.59	114.54	88.00
Gpx7	11.70	22.21	63.85	65.37
Mfsd4b4	90.08	94.05	323.17	376.82
Isg15	150.16	113.80	527.42	392.05
Dtl	68.39	78.40	304.05	358.77
Cul9	434.97	514.00	1424.51	1566.33
Esco2	24.13	13.71	148.16	132.86
Birc5	35.69	24.34	207.19	186.03
P2ry12	4.35	7.00	44.90	37.43
Smco4	27.99	18.30	128.84	126.74
Stil	28.15	16.67	159.91	115.41
Aox4	7.27	2.07	50.74	43.12
2010300C02Rik	1.45	3.25	33.13	41.33
Klf5	2.92	3.25	39.08	34.85
Gm37780	84.78	91.04	372.90	605.02
Gm43990	6.25	8.06	49.72	58.15
Per3	235.19	253.39	769.62	868.18
Csf2rb2	15.03	8.56	85.29	68.79

Dctd	40.82	47.51	158.36	193.95
Rnf43	87.16	86.95	332.91	289.85
Scd2	1988.45	2051.37	6298.09	6086.62
Cd80	36.40	29.82	159.24	121.83
Bzw2	657.85	653.62	2043.82	2214.35
Fgl2	27.80	16.45	150.90	137.89
Trabd2b	8.30	16.40	69.09	65.47
Tjp2	60.60	85.49	226.10	256.30
Ltk	506.00	375.82	1557.18	1270.94
Tlcd2	28.15	32.84	113.48	99.40
Wwc1	3.42	3.75	36.05	32.39
Gm35419	1.00	0.56	29.45	28.05
Col15a1	0.50	1.51	29.48	29.46
Gpm6b	61.65	57.80	230.01	261.80
Mical3	475.18	538.70	1443.41	1614.93
Adam3	1.47	2.01	32.45	44.60
Hck	2503.43	2250.54	7571.84	6907.07
Mcoln3	260.37	118.14	917.05	535.97
Isoc2b	52.28	77.70	199.76	225.74
Cdca3	74.56	62.55	307.02	246.19
Shcbp1	23.73	12.25	127.62	111.22
Mfsd2a	13.24	23.39	67.20	96.32
Raph1	11.06	6.88	68.99	52.28
Hmmr	23.59	15.27	153.71	115.48
Heyl	1.45	1.01	32.14	34.15
Cdca2	63.28	60.43	235.02	171.99
Ikbke	627.32	625.03	1884.28	1751.89
Ypel2	322.75	193.65	1028.11	609.99
Nuf2	18.31	16.90	123.14	111.62
Mir704	12.72	14.83	63.37	63.19
Azin2	2.47	2.07	32.28	45.01
Abr	1098.41	1182.51	3340.30	3202.67
Prdm1	114.87	107.44	436.59	336.26
Nbl1	3.95	2.52	40.72	50.64
Cdk1	44.60	33.90	222.39	210.09
Crim1	164.83	344.33	510.68	737.14
Tbx19	1.95	0.56	31.71	33.63
Ttk	19.28	18.52	99.31	63.60
Plscr1	107.38	88.70	347.96	362.14
Akr7a5	480.52	512.25	1453.08	1223.96
Ephx2	1.47	3.69	29.31	61.16
Bid	1383.02	1230.30	4059.46	3821.72
Tcf19	73.06	62.94	294.14	270.41
Smad3	1011.58	975.90	3020.89	2928.70

Gm22720	7.80	3.58	58.14	74.93
Tyms	59.93	72.18	210.44	211.58
Matk	404.94	471.36	1182.19	1414.84
BC030867	5.30	4.76	46.99	44.92
Cdh5	18.57	19.58	83.17	80.62
6530402F18Rik	97.94	164.49	311.96	426.85
Nme4	12.60	15.39	58.50	115.78
Kif13a	123.72	195.99	382.62	500.55
Myl4	317.79	342.88	950.54	1063.43
Slc6a21	10.18	12.70	52.81	48.15
Tfpi	49.68	51.81	166.15	150.77
1700048O20Rik	85.22	61.09	267.10	378.76
Naip5	410.18	419.32	1237.00	1123.76
Uhrf1	238.72	189.84	770.72	653.17
Poglut2	73.32	103.95	232.32	261.44
Tet1	80.67	60.66	281.03	296.34
Cdk5r1	334.29	361.62	960.59	964.85
Stxbp1	73.82	80.96	233.14	276.61
Pla2g4a	20.37	21.37	85.19	114.83
Scamp1	5.82	6.10	46.86	50.63
Gm17999	1.00	2.69	25.29	37.96
Bcl2l15	0.97	3.08	27.43	26.79
Nusap1	96.49	72.06	391.05	351.72
Odaph	6.82	12.20	41.36	60.48
Nlrx1	57.20	83.70	185.48	244.70
Ankle1	12.46	11.02	108.17	93.24
Zfp239	99.67	102.59	336.38	413.65
Ly6k	46.85	62.01	177.02	214.87
Rrm2	120.94	102.44	457.27	469.92
Dnaja4	8.44	7.16	79.18	114.47
9430041J12Rik	0.95	1.00	27.38	29.90
Chil6	0.48	1.00	24.89	30.45
Lmbr1	21.30	22.44	85.29	78.95
Rgmb	90.40	97.81	273.09	277.37
Gng7	3.40	1.68	31.68	47.62
C030013C21Rik	8.27	9.06	45.62	61.39
Pqlc2	664.63	608.69	1826.83	1563.34
Mettl21a	115.35	93.66	335.63	324.66
Zfp976	31.73	33.74	116.24	142.55
Tmem151b	31.03	24.68	112.40	113.47
Slc29a3	1062.94	921.22	2910.89	2522.60
Tfdp1	302.65	302.22	889.98	922.60
Chn2	14.05	9.18	66.28	71.82
Cdh23	5.35	6.44	39.98	28.54

Tmem231	61.67	60.54	189.76	226.40
Zfp442	8.84	11.25	48.39	40.06
Gm40634	18.92	22.16	77.77	76.49
Tmeff1	9.29	13.04	51.87	56.44
Melk	14.46	9.40	86.48	61.92
Myc	6752.05	10081.41	24389.19	26413.79
Plxnc1	2779.47	2709.26	7459.99	6708.34
Zfp612	27.86	33.74	96.27	135.40
Slc4a8	391.57	392.40	1102.12	1104.55
Lacc1	186.48	222.39	526.40	728.36
Pbk	9.61	8.34	71.56	60.04
Ckap2l	40.27	30.05	170.68	158.36
Gm26560	1.97	0.50	29.59	17.19
Mirt1	3060.66	2579.91	8193.26	7402.73
1110002J07Rik	0.97	1.51	28.34	21.41
Snhg18	55.36	55.50	178.73	197.80
Rtl8c	2.47	1.57	28.54	33.48
5930403N24Rik	1.00	2.07	26.85	27.31
Igkv1-108	8.18	2.24	51.25	50.54
Camk2a	35.90	43.14	119.28	116.43
Naip6	265.92	165.47	741.37	571.94
Clstn1	123.55	141.05	347.64	431.43
Ptprs	447.57	454.79	1222.95	1116.04
Robo1	1.50	1.01	26.19	42.07
Megf8	27.38	32.96	97.15	84.22
Col20a1	108.26	75.70	305.35	377.11
E2f1	139.62	124.56	394.73	425.61
Gm50462	0.50	1.00	23.96	26.31
Slc35f2	43.79	38.77	137.60	143.75
Prr11	28.10	21.09	121.05	91.18
Ncapg	56.40	33.85	225.98	220.16
Gm28417	96.07	111.94	283.23	375.55
4930451G09Rik	69.42	55.94	212.32	177.69
Grik5	67.30	64.74	201.47	244.08
Plpp3	284.81	261.18	766.98	734.27
Acot6	11.31	9.51	53.43	54.22
Plscr4	2.92	0.56	30.55	28.27
Rilpl2	1694.23	1831.22	4610.69	4947.39
Nucb2	445.46	509.61	1168.83	1205.54
Anln	25.11	16.11	114.37	87.11
Rnasel	425.20	449.71	1136.46	1248.14
Col6a6	5.42	7.05	54.18	64.36
Poglut3	6.32	3.69	35.90	40.54
Ptgr1	11.99	10.18	66.95	59.25

Gm11998	163.87	77.76	512.42	329.03
Jdp2	19.02	23.17	71.16	101.25
Slc25a13	15.98	15.89	64.41	80.51
2900026A02Rik	13.05	33.90	55.61	167.28
Trim32	104.16	99.59	297.92	275.72
Gm26574	5.89	5.26	35.54	43.10
Tyrobp	465.86	392.41	1221.62	1045.60
Zfp937	19.47	15.05	71.07	80.62
Fbxo10	66.52	83.70	191.58	194.24
Gm12679	8.20	6.66	48.67	56.18
Rasl11a	5.33	3.13	34.74	47.24
Hes1	1062.14	1134.24	2769.89	3278.48
Cacna1e	4263.80	7272.93	10833.29	14981.43
Cdca8	85.25	85.05	268.35	216.81
Hspa12a	194.27	123.81	522.22	394.16
Ube2r2	2009.02	2006.25	5193.06	5028.37
Mtln	75.58	108.59	218.78	254.32
Prkcz	79.34	51.93	245.76	252.14
Edaradd	1315.66	1204.23	3420.32	3128.45
Lrtm2	11.53	15.95	55.75	56.62
Odc1	1595.12	2041.60	4334.96	4820.11
Gramd1b	516.76	537.90	1324.77	1434.59
Nmrk1	197.81	189.84	515.70	471.41
Bsn	56.61	56.51	172.62	175.36
Troap	17.50	7.39	70.44	66.02
2210414B05Rik	32.34	25.23	108.19	121.76
Myo10	67.26	51.75	210.26	173.31
Erich2	3.35	1.00	33.21	16.19
Traf1	1697.98	1833.77	4367.39	4626.43
Neil3	18.64	10.13	125.26	99.43
Arsb	286.98	283.13	736.93	678.08
Tmem18	260.00	259.95	683.11	727.94
Rad54b	21.80	18.58	85.10	62.00
Rad54l	69.08	61.43	211.62	209.76
Cblc	5.26	7.39	44.16	46.69
Lpcat2	317.12	439.33	841.53	976.48
Cipc	1205.43	1196.28	3002.07	3051.50
Car9	7.77	13.32	38.17	43.13
Acad8	73.83	57.35	208.78	168.11
Borcs7	276.54	256.13	710.75	704.09
Gm6377	2143.73	2599.47	6891.57	7411.19
Gm20069	1.00	1.00	24.95	24.40
Obscn	33.68	18.70	127.75	86.72
Grhpr	415.54	427.45	1038.71	969.07

Ankrd13b	13.98	14.55	59.32	54.10
Morn1	38.78	33.85	114.79	136.24
Gask1a	14.26	7.89	60.63	67.21
Prag1	1136.81	1044.53	2946.55	2507.39
Armc2	10.18	9.57	46.35	40.42
Psap	14171.19	13832.63	34476.83	33472.35
Gm6637	6.35	5.26	33.83	63.16
Ak7	3.90	9.51	26.19	47.16
Cit	111.19	96.39	323.87	294.22
Rgs3	348.57	297.07	871.84	705.86
Grk5	243.45	229.48	614.43	671.99
Abcg3	579.93	456.74	1419.71	1213.58
Fam228b	10.65	10.69	44.82	49.18
Kif4	37.23	21.88	149.77	145.30
Echdc3	20.83	19.53	70.75	67.81
Kif2c	22.04	10.52	120.73	77.33
Slc20a2	669.48	654.41	1619.64	1466.39
Ctsb	2366.74	2561.84	5647.73	5725.78
Kif23	236.85	192.20	636.43	480.20
Bcl2a1b	91.10	109.71	244.55	316.21
Myzap	200.93	210.21	497.03	487.67
Kif15	101.28	76.09	331.21	287.54
Cdca7l	150.56	119.96	388.37	517.73
Plbd1	1037.65	1604.69	2488.58	3472.51
1700027J07Rik	46.29	25.06	151.01	161.11
Gm12493	5.75	4.08	39.80	47.59
Hpgds	308.63	272.82	775.41	606.12
Gm37145	46.10	50.24	133.91	137.64
Prkar2a	263.18	295.89	664.32	679.64
Plk1	44.72	42.08	146.08	121.81
Tnfsf18	6.78	6.38	33.58	43.03
Cenph	38.25	38.94	118.14	123.29
9530020I12Rik	1.43	1.12	23.30	34.87
Msl3l2	31.71	27.42	104.81	83.50
Mki67	465.46	263.15	2558.08	2105.36
Kif14	20.35	4.31	95.82	88.21
Rnd3	8.75	11.64	35.99	80.10
Top2a	384.97	249.89	1915.98	1466.68
Acer2	8.84	10.74	39.52	60.73
Cntln	39.63	40.17	122.16	125.04
Oosp2	0.48	0.50	21.17	26.36
Gm34829	1.00	3.25	23.55	51.08
Tmtc2	2.42	0.56	23.73	20.24
Pimreg	5.75	2.69	42.89	36.19

Aurkb	160.61	136.18	435.96	340.21
Ighv6-3	416.00	429.15	1002.33	969.69
Ccnb2	56.18	50.47	175.69	155.58
Pacc1	861.76	575.83	2016.53	1656.64
Sbf2	254.32	195.84	612.85	555.42
Serpina3g	828.42	1428.09	1990.89	3400.58
Pik3r3	59.77	54.83	158.23	169.64
Nipal4	1.00	3.81	21.55	36.21
Tspear	22.42	17.51	68.40	78.07
Gm12504	20.42	24.12	67.84	98.27
Rab37	227.81	185.62	573.93	676.03
Tmem41a	29.67	24.73	86.50	89.52
Bcl7a	2534.02	2592.76	5952.91	5454.67
Antxr1	4.37	2.13	27.18	21.79
Cks1b	57.82	56.40	172.01	153.12
Prc1	96.00	66.75	286.03	275.47
C430002N11Rik	15.43	8.89	55.58	70.03
Grhl1	33.92	26.02	103.07	90.46
C030013G03Rik	0.97	0.56	19.71	16.74
Mzt2	61.24	43.25	158.47	167.95
Skida1	2.92	5.09	26.53	32.93
Rnft2	24.55	11.13	76.02	77.02
Src	8.27	11.64	34.51	50.64
Tmem154	890.36	729.87	2721.97	2353.80
Cenpe	143.92	107.93	406.21	299.12
Zfp992	27.06	21.43	85.65	65.68
As3mt	430.71	421.68	1002.40	1025.06
Anxa9	63.87	61.89	166.22	173.36
Abhd17c	441.57	428.53	1018.58	1011.32
Aspm	75.34	60.26	242.54	179.84
Mdfic	507.60	551.03	1167.55	1230.20
Bcl2a1d	25.22	43.63	78.94	115.09
Ccnf	113.90	80.41	289.74	255.10
Hgfac	1.00	1.00	17.87	33.06
Zfr2	45.26	39.00	121.41	128.39
Mapre3	60.23	75.25	156.42	196.89
Slc7a7	154.16	139.39	375.61	305.78
Smim10l1	319.58	316.84	735.11	758.88
Prob1	105.08	88.96	255.41	251.51
Tspan31	369.35	368.13	865.68	828.74
Actrt3	2.47	8.45	23.08	40.69
Rundc3a	23.42	10.13	71.84	54.65
Gbp7	564.06	579.13	1329.99	1422.09
Pyroxd2	42.03	38.38	114.48	99.40

Tenm4	120.31	97.97	285.97	228.72
Zfp783	309.89	381.13	713.23	1016.64
Slc29a1	327.05	331.31	757.75	835.27
Klk8	4.85	20.42	26.31	69.53
6330562C20Rik	14.78	14.21	55.95	49.17
Btbd19	286.26	309.01	657.51	728.29
Fhit	15.05	13.43	51.42	45.64
Golim4	233.62	214.12	540.52	517.15
Arhgef9	3.88	9.01	25.82	40.76
Hcst	294.53	295.52	678.33	653.55
Gmnn	160.17	146.35	386.55	517.39
Zscan12	71.78	59.97	182.98	199.30
Pif1	23.37	10.46	96.33	65.15
F730043M19Rik	114.92	97.60	311.66	318.44
Lmo7	26.15	8.45	88.89	62.00
Spc24	35.18	35.25	122.83	126.67
Cenpm	20.26	24.45	68.43	71.90
Fbxw17	159.49	149.42	380.03	526.86
Trim6	9.63	28.59	37.52	89.28
Wdfy2	2528.74	2687.37	5665.13	6154.06
Zfp975	38.25	36.93	102.72	99.85
Zdhhc1	107.04	110.90	255.98	285.10
Pip4k2a	3438.55	3492.12	7700.52	7821.81
Ptprv	175.13	155.31	402.52	410.14
Fam83h	8.70	6.77	36.72	37.48
Gm45206	32.37	34.47	92.53	101.43
Phf19	362.15	394.54	819.35	834.58
Rps6ka1	1607.53	1494.36	3577.51	3102.07
Gm28802	9.20	10.40	37.20	46.09
Wdr60	62.25	54.50	159.11	158.64
Sema4b	4146.95	3791.79	9034.39	8488.48
Sapcd2	5.85	5.93	41.24	40.16
Kif11	87.17	51.09	556.33	433.83
Slc25a4	1337.97	1479.30	2908.43	3286.13
Gm13986	1.00	0.56	16.82	23.42
Rogdi	1290.22	1167.72	2809.39	2905.37
Egr2	405.89	624.67	955.73	1423.27
Dna2	137.94	94.39	324.99	308.30
Atp11a	18.97	15.44	56.57	105.77
Selenbp1	17.93	23.44	59.01	70.72
Gpr68	18.00	32.84	55.73	86.55
Syngap1	45.72	32.18	117.96	92.94
Ralb	209.70	238.80	466.29	511.64
Cdc6	36.56	30.72	115.42	144.71

Thyn1	104.33	79.17	245.68	235.85
Ahcyl2	1074.91	1097.81	2334.39	2814.55
Gm37759	8.73	3.81	36.67	26.83
Lynx1	2021.00	2214.07	4528.52	5716.50
Pld4	6574.94	5366.23	14098.68	11507.84
Rabl2	37.58	45.10	97.07	108.66
Nlrc3	1317.61	1278.04	2841.59	2900.81
Slc43a1	202.11	126.97	488.88	408.23
2010016I18Rik	248.02	257.63	549.16	618.15
Slc36a4	150.79	123.95	352.00	348.93
Spns3	497.46	481.75	1083.96	1023.19
Copg2	1088.80	1157.25	2342.00	2421.08
Gm50386	170.43	119.23	375.06	409.23
Ccr5	78.80	68.15	185.71	177.13
Diaph3	49.20	48.51	133.83	155.44
Stmn1	51.13	51.65	142.49	138.49
Kif5a	7.30	8.06	31.57	43.15
Capn2	522.76	421.29	1121.88	947.85
Espn	4.87	3.86	27.66	24.83
Pde1c	17.95	21.99	53.86	73.54
Arhgap27os3	799.95	861.47	1792.54	3014.09
Ticrr	94.71	81.40	232.58	212.94
Ndst1	4721.55	4140.83	9989.16	9695.28
Atp6v1g2	72.82	75.20	168.30	186.49
Klrb1f	44.62	32.79	110.40	123.44
A730063M14Rik	22.78	20.42	64.16	88.19
Dnhd1	233.04	229.01	524.00	664.88
Armc3	99.28	105.85	223.73	234.44
Bbs5	6.85	4.76	28.79	34.01
Sfmbt2	2.90	3.13	21.21	31.00
Nsg2	10.65	9.96	39.05	37.96
Serpinb6b	75.35	85.82	184.74	262.06
Ank3	0.97	2.69	17.98	25.98
St3gal4	69.99	66.14	164.10	162.81
Parpbp	21.28	16.33	79.38	65.65
1700034H15Rik	12.72	14.10	44.48	50.64
Rcn1	163.02	178.15	355.12	430.78
4930581F22Rik	110.04	100.55	245.42	249.12
Arhgef39	37.33	40.95	93.86	101.79
Tmcc3	517.58	451.69	1146.91	1107.26
Kctd17	1.50	3.75	18.35	29.42
Slc43a3	174.16	166.10	433.18	454.75
Tmed6	5.37	6.77	26.39	37.45
Tgm2	27.01	26.58	74.71	115.23

Abhd5	71.51	53.49	189.79	198.64
Gm44260	99.09	79.18	223.55	213.63
Car7	34.73	39.39	86.95	97.23
Gm34121	13.12	7.72	44.76	47.45
Slc12a5	23.42	25.73	69.01	200.18
Ccdc116	80.78	87.28	178.72	201.60
Mccc2	300.52	255.49	646.22	635.96
Plekhg5	56.82	62.39	136.80	155.70
Tlr7	1914.72	1267.31	4141.90	3502.47
Gm28913	10.74	6.44	36.61	48.20
Fanca	779.97	653.36	1613.78	1746.39
Sfxn1	918.12	977.55	1890.47	2213.18

Supp Table 4				
**Gene**	**WT FO**	**cKO FO**	**WT MZ**	**cKO MZ**
Gm4734	3.42	4.75	2.87	356.38
Ngp	12.81	4.30	13.86	140.38
Ltf	5.44	6.09	3.83	94.52
Adgrd1	12.74	4.19	39.17	45.05
Smox	10.82	9.45	36.68	73.92
Specc1	18.38	21.53	52.70	122.76
Pax6	3.92	6.88	7.17	137.56
Sh2d1b1	7.71	2.62	32.85	46.94
Gm48505	20.33	16.44	56.57	83.08
Adgra2	20.42	23.83	45.53	103.17
Art3	8.27	2.01	27.90	30.88
Kif22	116.96	56.78	283.49	257.23
Camp	6.40	3.57	8.53	53.04
Snca	1.95	4.58	4.28	72.38
Gm5915	6.40	3.69	3.83	53.43
Gm15796	4.97	4.25	10.49	77.13
Gm5236	1.00	2.62	1.48	100.49
Atp2b4	12.08	6.43	14.96	57.73
Aqp1	9.68	15.49	10.68	110.12
Spire1	6.30	8.33	27.50	53.88
Gpr34	25.45	58.06	161.99	534.53
Rad51b	16.59	10.90	36.82	55.63
Ttc41	59.62	50.74	129.66	164.98
Poln	44.13	33.23	86.31	116.07
Nrep	9.32	8.83	20.88	101.90
Add2	1.97	4.19	8.14	43.65
Serpina3e-ps	3.90	6.20	2.90	93.23
Prom1	7.32	3.74	15.10	34.82
Gm46611	19.66	28.08	18.72	172.81
Tspan33	5.37	3.63	6.94	46.08
Trps1	635.81	540.50	1297.19	1516.94
Eno2	12.67	9.89	17.91	72.35
Timd2	10.65	1.00	40.04	33.22
Ccdc122	35.45	39.60	83.76	134.06
Fn1	51.87	15.05	57.81	103.37
Sptb	22.92	26.29	38.57	132.65
Otx2	1.97	3.74	1.96	64.88
Mpo	2.47	9.39	4.28	78.35
Epb42	6.40	7.66	4.71	69.94
Znrf3	77.34	61.38	152.00	180.28
Rad51	75.87	51.07	175.24	186.75
Strip2	2.93	9.89	12.01	53.14

Asns	12.27	14.04	36.54	65.99
Rnase4	13.98	8.00	47.54	52.01
Car1	20.47	14.93	14.76	84.24
Aldoart2	1.50	2.06	3.35	80.40
Gm17025	3.45	4.19	13.15	32.69
Gm14892	1.00	1.00	1.93	101.54
Optn	214.44	156.94	438.95	415.21
Chil3	17.86	3.74	9.27	61.44
Fggy	35.66	31.72	80.96	97.91
3000002C10Rik	47.25	41.23	88.94	137.98
Cdc45	107.10	84.03	252.54	261.20
Alox5ap	42.77	40.95	85.95	124.42
Zkscan7	202.95	166.72	423.58	431.68
Ermap	30.90	26.34	36.74	107.96
Nbdy	33.90	35.97	72.16	113.63
Dclre1a	63.98	46.21	140.45	134.34
Dmtn	1.00	2.57	4.28	35.01
Gm43065	31.76	19.47	67.26	73.02
Ggnbp1	37.54	42.90	68.92	127.79
Ncapd3	703.61	524.10	1371.00	1294.35
Sgip1	4.38	4.13	21.07	32.16
Rorb	2.97	1.50	5.28	40.11
Oip5	26.46	20.25	45.14	69.84
Ank1	23.30	42.68	49.67	141.49
Fam135a	8.73	2.51	23.06	26.35
Gm15523	1.50	1.00	15.58	22.72
Gm11942	29.36	40.95	49.08	140.08
Chaf1a	122.20	110.65	292.02	332.77
Gm43071	212.31	185.49	448.00	461.94
Cip2a	138.89	97.47	288.36	266.97
Rps19-ps4	3.42	6.26	1.93	45.73
Nek2	59.70	41.45	138.49	157.74
Trim10	3.88	6.65	4.28	76.29
Zmat4	1.48	1.56	15.67	24.41
Lmntd1	12.60	9.06	34.89	41.16
Zfp985	7.30	10.23	23.06	44.62
Ypel4	3.38	5.25	5.19	42.07
Dmd	25.87	19.53	71.78	71.48
Gm16216	2.45	3.74	5.19	38.53
Car12	3.42	1.56	17.11	22.84
Pon3	50.97	48.17	116.71	136.66
Slc35g1	11.74	12.08	39.82	55.20
Zfp518b	106.19	79.90	184.61	201.19
Rad51ap1	68.68	47.61	153.88	146.45

Gm12726	3.45	1.50	7.09	34.98
Stk39	4.40	4.25	18.16	27.28
2900005J15Rik	8.80	11.18	27.67	44.87
9530062K07Rik	20.31	15.71	38.80	56.23
Soat2	5.30	2.57	19.77	26.28
C920021L13Rik	121.02	97.62	257.92	260.22
Hk3	30.15	28.14	64.45	83.80
Myo3b	20.56	13.59	45.82	48.21
BC147527	184.90	125.08	575.32	552.69
Nova1	1.48	1.00	9.10	29.70
Dennd2a	4.85	2.57	18.81	22.84
Cryl1	208.74	188.87	404.50	451.77
Gm11703	1.50	3.01	1.48	55.10
Gm7312	1.97	3.07	3.35	45.84
Zranb3	219.37	169.64	406.67	400.67
Cbx2	71.96	46.21	153.26	122.24
Cyb5r2	2.47	2.68	14.81	26.79
Med12l	87.25	62.66	187.63	159.28
Evi2a	1044.12	1058.72	2143.26	2429.96
44256	17.02	26.62	41.66	86.55
Gm9159	5.40	7.38	5.22	62.83
1700029J07Rik	390.04	344.87	793.28	782.01
E230016M11Rik	99.87	67.92	172.11	170.98
Mest	57.29	57.56	117.08	159.62
Akap17b	164.79	102.05	308.30	252.02
Enah	9.73	5.14	13.57	31.66
Zfp786	5.35	4.08	23.94	25.85
Apof	48.27	35.12	107.57	113.41
Gm4977	16.17	18.29	24.02	247.62
Myo1b	5.92	9.11	17.31	38.60
Gm5879	2.45	5.87	6.10	35.87
Aifm2	20.90	36.13	54.99	98.20
Ncapg2	148.06	96.66	342.79	294.06
Dnph1	2.90	2.57	18.04	27.67
Kel	1.95	4.13	3.26	57.35
Fuca2	573.46	515.63	1193.30	1151.31
Ube2e3	351.82	300.83	694.30	672.39
Tmc1	15.71	11.52	48.41	47.09
44440	5.85	4.13	8.87	26.18
Dennd2d	3154.38	2945.35	6269.47	6419.64
Depdc1b	6.33	6.88	27.75	34.24
Mst1	153.22	156.53	328.55	358.97
Cd180	6455.01	7296.71	12912.29	16008.75
Ska3	33.21	22.38	62.53	68.00

Mageh1	7.80	8.78	22.49	36.08
Lgalsl	31.52	24.22	69.91	78.71
Ceacam16	450.12	378.71	819.22	828.97
Gm32401	276.05	228.60	510.50	517.61
Gda	9.16	10.23	11.64	65.00
Gm12739	2.93	4.81	3.41	49.91
Gm12912	12.58	18.29	23.60	346.64
Klrg1	6.28	2.57	12.18	22.33
Tctex1d2	95.80	79.15	218.67	195.71
Zwilch	128.85	96.56	240.16	227.33
Inca1	29.31	19.58	55.17	57.09
Gm37607	42.63	27.41	68.52	79.43
Apobr	25.61	18.96	61.59	58.36
Dram2	982.37	845.01	1852.98	1816.60
Cryzl2	138.22	157.28	258.58	350.75
Gm35315	46.23	26.40	92.50	87.94
Pam	7.35	6.71	22.91	30.52
Gm43672	132.61	122.06	211.54	282.42
Ppfia4	38.47	28.24	81.88	80.76
Morrbid	9.73	9.89	34.08	39.00
Zfp248	21.47	28.58	54.89	76.06
Ccdc162	4.88	5.25	19.72	25.33
Nol4l	16.71	16.56	50.69	56.61
Nckap1	8.30	7.77	15.70	39.87
Tssk4	34.50	29.75	77.76	80.00
Adam15	101.38	91.36	172.77	216.25
Fscn1	9.77	10.29	30.73	41.02
Gm6749	1.50	2.06	1.96	30.34
Cdc25c	14.93	9.50	51.08	41.30
Extl2	74.32	54.94	164.60	141.38
Gm19345	139.60	117.48	278.71	262.32
S100a9	14.33	15.37	20.34	316.92
Smim24	63.12	59.92	144.41	143.04
Igfbp5	3.40	2.51	6.72	37.00
Smad6	1.50	1.56	17.54	19.29
Ccnb1	36.02	21.31	60.65	72.32
Meis1	11.70	15.55	13.63	66.75
4930426D05Rik	1248.44	1120.60	2411.38	2348.34
Entpd1	415.17	433.65	867.22	940.61
AU020206	5213.93	5153.65	10420.42	10767.02
Catsperg1	39.13	21.42	46.93	60.79
Fancd2	101.02	84.92	229.61	207.68
Fsd2	33.52	34.45	73.88	89.11
Insyn1	4.42	2.62	21.79	20.30

Pfn2	64.04	46.10	67.03	124.05
Fads6	4.40	2.62	19.09	19.29
Cyp2u1	5.42	6.76	23.51	28.87
Gm12988	3.95	4.13	8.93	41.18
Pbx4	152.64	131.37	304.33	285.95
Cd59b	1.00	6.20	14.48	28.91
Mcrip2	20.42	17.34	34.18	50.61
Prr22	37.85	35.64	72.35	89.54
Serpinb9	119.97	129.41	235.20	297.87
Tmcc2	54.16	42.79	39.90	277.02
Dapk2	2.97	1.56	8.96	19.11
St14	837.43	942.43	1688.54	1964.77
Tbc1d24	39.06	40.62	73.31	98.80
Gm18119	5.40	6.26	5.70	41.15
Gpr179	5.87	7.21	24.16	30.44
F11r	4.97	3.07	23.06	22.81
Kif20a	103.08	93.16	238.20	234.23
Wdr6	1642.58	1468.92	3224.44	3075.50
Bub1b	181.10	147.12	432.24	373.28
M1ap	36.42	37.37	63.88	92.41
Gm50253	4.88	4.75	17.34	24.46
Gm10644	46.79	32.56	74.67	80.71
Nlgn2	5.33	8.94	27.90	39.34
Aldh1l2	24.25	14.82	40.83	46.01
Gm44250	32.05	33.11	80.70	84.67
Lcn2	3.99	5.14	2.36	49.92
Pglyrp2	872.99	862.41	1650.21	1775.18
